# Knockdown of SOX2OT inhibits the malignant biological behaviors of glioblastoma stem cells via up-regulating the expression of miR-194-5p and miR-122

**DOI:** 10.1186/s12943-017-0737-1

**Published:** 2017-11-13

**Authors:** Rui Su, Shuo Cao, Jun Ma, Yunhui Liu, Xiaobai Liu, Jian Zheng, Jiajia Chen, Libo Liu, Heng Cai, Zhen Li, Lini Zhao, Qianru He, Yixue Xue

**Affiliations:** 1 Department of Neurobiology, College of Basic Medicine, China Medical University, Shenyang, 110122 People’s Republic of China; 2 Key Laboratory of Cell Biology, Ministry of Public Health of China, and Key Laboratory of Medical Cell Biology, Ministry of Education of China, China Medical University, Shenyang, 110122 People’s Republic of China; 3 Department of Neurosurgery, Shengjing Hospital of China Medical University, Shenyang, 110004 People’s Republic of China; 4 Liaoning Research Center for Translational Medicine in Nervous System Disease, Shenyang, 110004 People’s Republic of China; 5 Key Laboratory of Neuro-oncology in Liaoning Province, Shenyang, 110004 People’s Republic of China

**Keywords:** SOX2OT, miR-194-5p, miR-122, SOX3, TDGF-1, Glioma

## Abstract

**Background:**

Accumulating evidence has highlighted the potential role of long non-coding RNAs (lncRNAs) in the biological behaviors of glioblastoma stem cells (GSCs). Here, we elucidated the function and possible molecular mechanisms of the effect of lncRNA-SOX2OT on the biological behaviors of GSCs.

**Results:**

Real-time PCR demonstrated that SOX2OT expression was up-regulated in glioma tissues and GSCs. Knockdown of SOX2OT inhibited the proliferation, migration and invasion of GSCs, and promoted GSCs apoptosis. MiR-194-5p and miR-122 were down-regulated in human glioma tissues and GSCs, and miR-194-5p and miR-122 respectively exerted tumor-suppressive functions by inhibiting the proliferation, migration and invasion of GSCs, while promoting GSCs apoptosis. Knockdown of SOX2OT significantly increased the expression of miR-194-5p and miR-122 in GSCs. Dual-luciferase reporter assay revealed that SOX2OT bound to both miR-194-5p and miR-122. SOX3 and TDGF-1 were up-regulated in human glioma tissues and GSCs. Knockdown of SOX3 inhibited the proliferation, migration and invasion of GSCs, promoted GSCs apoptosis, and decreased TDGF-1 mRNA and protein expression through direct binding to the TDGF-1 promoter. Over-expression of miR-194-5p and miR-122 decreased the mRNA and protein expression of SOX3 by targeting its 3’UTR. Knockdown of TDGF-1 inhibited the proliferation, migration and invasion of GSCs, promoted GSCs apoptosis, and inhibited the JAK/STAT signaling pathway. Furthermore, SOX3 knockdown also inhibited the SOX2OT expression through direct binding to the SOX2OT promoter and formed a positive feedback loop.

**Conclusion:**

This study is the first to demonstrate that the SOX2OT-miR-194-5p/miR-122-SOX3-TDGF-1 pathway forms a positive feedback loop and regulates the biological behaviors of GSCs, and these findings might provide a novel strategy for glioma treatment.

**Electronic supplementary material:**

The online version of this article (10.1186/s12943-017-0737-1) contains supplementary material, which is available to authorized users.

## Background

Glioma is the most common primary malignant tumor of the brain, and the median survival time is less than 12 months [1, 2]. At present, glioma treatment involves surgery, chemotherapy and radiotherapy. GBM is highly invasive and migratory, leading to frequent relapse after operation, with a short survival time [3–5]. Glioblastoma stem cells (GSCs) are undifferentiated glioma cells, and are related to chemotherapy and radiotherapy resistance, and the poor prognosis of glioma [6]. With the progress in genetic and molecular studies, an increasing number of scholars consider GSCs to be target cells for glioma therapy [7].

Long non-coding RNAs (lncRNAs) are a kind of non-coding RNAs (ncRNAs) longer than 200 nucleotides. Although lncRNAs do not encode proteins, they are key participants in a variety of biological processes, including chromatin remodeling, alternative splicing, and mRNA stability [8–10]. Research in recent years has accumulated evidence that lncRNAs can act as oncogenes or tumor suppressors, and are closely related to the tumor occurrence and development [11]. For example, lncRNAs, such as HOTAIR, CRNDE, GAS5 and other lncRNAs with abnormal expression in glioma tissues and cell lines, regulate the biological behaviors of glioma cells [12–14]. SOX2OT is a lncRNA that is mapped to the human chromosome 3q26.3 (Chr3q26.3) locus [15], and is highly expressed in colorectal cancer, lung cancer, breast cancer and esophageal squamous cell carcinoma. Moreover, it is positively correlated with the proliferation, migration and invasion of tumor cells [16–19]. Knockdown of SOX2OT in lung cancer inhibited cell proliferation by inducing G2/M arrest. In gastric cancer, hepatocellular carcinoma and lung cancer, SOX2OT expression was positively associated with histological grade and TNM stage, which are significantly associated with overall survival and poor prognosis of patients as independent prognostic factors [20, 21]. However, to the best of our knowledge, the clinical significance of lncRNA SOX2OT in glioma tissues remains unclear.

MicroRNAs (miRNAs) are kind of single-stranded ncRNAs approximately 22 nucleotides long. MiRNAs usually bind to partially complementary binding sites typically located in the 3′ untranslated region (UTR) of target mRNAs and degrade target mRNAs, thus repressing their expression [22, 23]. Several studies have shown that miRNAs can act as oncogenes or tumor suppressor genes in tumors, and treatment that target miRNAs have been widely studied in a variety of tumors [24–26]. The expression level of miR-194-5p is markedly decreased in gallbladder cancer cells, and over-expression of miR-194-5p markedly promoted cells into S-phase and cell apoptosis, which suggested that miR-194-5p acts as a tumor suppressor gene in gallbladder carcinoma tissue [27]. However, the relationship between miR-194-5p and glioma is still unclear. Moreover, miR-122 act as a tumor suppressor gene in breast cancer [28]. Abnormal expression of miR-122 in primary tumors appears to play important roles in the development of colorectal liver metastasis [29], and miR-122 can remarkbly inhibit the growth of hepatocellular carcinoma through down-regulation of the target gene MEF2D [30]. MiR-122 is under-expressed in glioma tissues and glioma cell lines, and the expression level of miR-122 is correlated with patient survival. Moreover, miR-122 over-expression can suppress the proliferation, migration and invasion of glioma cells [31].

SOX3 is a transcription factor that belongs to the SOX family. The SOX3 gene maps to chromosome Xq27, which is one of the earliest neural markers in vertebrates [32]. SOX3 acts as a key regulator of biological behavior in a variety of cells, including the development of pituitary and testis [33, 34]. SOX3 is highly expressed in esophageal squamous cell carcinoma, and is associated with poor prognosis [35]. The relationship between SOX3 and glioma has not been reported.

Cripto-1 (CR-1)/teratocarcinoma-derived growth factor1 (TDGF-1) is a member of the epidermal growth factor (EGF)-Cripto FRL gene family. It was initially isolated from teratocarcinoma cells, and regulates cell differentiation and early embryonic development [36]. TDGF-1 showed low expression in normal tissues and cells, while it is highly expressed in colorectal cancer, gastric cancer, breast cancer, testicular cancer and other malignant tumors, and plays an important role in the process of tumor invasion and metastasis [37–40]. Previous studies have shown that the expression of TDGF-1 is up-regulated in glioblastoma multiforme tissues and blood, and is significantly positively correlated with shorter survival time in cancer patients [41].

In this paper, we studies the endogenous expression of SOX2OT, miR-194-5p, miR-122, SOX3 and TDGF-1 in GSCs, and their effects on the biological behavior of GSCs. Further, we explore whether SOX2OT can regulate the expression of SOX3 by regulating the expression of miR-194-5p and miR-122, and affect the biological behavior of GSCs. We also investigated the molecular mechanisms by which SOX2OT exerts its effects. Our results will suggest that SOX2OT might be a new molecular targets for the treatment of glioma.

## Methods

### Cell culture and human tissue samples

Human astrocyte (HA) cells were purchased from ScienCell Research Laboratories (Carlsbad, CA, USA) and grown in RPMI-1640 culture medium (Gibco, Grand Island, NY, USA) with 10% fetal bovine serum (FBS, Gibco, Carlsbad, CA, USA). Human glioma cell lines (U87 and U251) and human embryonic kidney (HEK) 293 T cells were purchased from Shanghai Institutes for Biological Sciences Cell Resource Center, and grown in Dulbecco’s modified Eagle medium(DMEM)/high glucose with 10% FBS. All cells were maintained in a humidified incubator at 37 °C with 5% CO_2_. Human glioma tissues and normal brain tissues (NBTs) were collected from patients at the Department of Neurosurgery of Shengjing Hospital of China Medical University (*n* = 5). All the tissue samples were immediately frozen in liquid nitrogen after surgical resection, and stored at −80 °C until use. Informed consent was obtained from all patients and the study was approved by the Ethics Committee of Shengjing Hospital of China Medical University. Glioma tissue samples were classified into five groups according to the 2007 WHO classification by neuropathologists: Grade I (*n* = 5), Grade II (n = 5), Grade III (*n* = 8) and Grade IV (n = 8).

### Isolation of GSCs

GBM stem cells (GSC-GBM) were isolated from GBM tissues according to the method described previously [42, 43]. GSC-U87, GSC-U251 and GSC-GBM were resuspended in DMEM/F-12 medium (Life Technologies Corporation, Grand Island, NY, USA) supplemented with basic fibroblast growth factor (bFGF, 20 ng/ml, Life Technologies Corporation, Carlsbad, CA, USA), epidermal growth factor (EGF, 20 ng/ml, Life Technologies Corporation, Gaithersburg, MD, USA) and 2% B27 (Life Technologies Corporation, Grand Island, NY, USA).

### RNA extraction and quantitative real-time PCR (qRT-PCR)

Total RNA was isolated from cells with Trizol reagent (Life Technologies Corporation, Carlsbad, CA, USA). RNA concentration and quality were determined via 260/280 nm absorbance with Nanodrop Spectrophotometer (ND-100, Thermo, USA). One-Step SYBR PrimeScript RT-PCR Kit (TakaraBio, Inc., Japan) was used to detect the expression of SOX2OT using 7500 Fast RT-PCR System. TaqMan MicroRNA Reverse Transcription kit (Applied Biosystems, Foster City, CA, USA) was used for the reverse transcription of miR-194-5p and miR-122, and the expression of miR-194-5p and miR-122 were detected with TaqMan Universal Master Mix II. GAPDH and U6 were used as the endogenous control. The expression levels were normalized to those of the endogenous controls, and fold changes were calculated using the relative quantification (2^−*ΔΔ*Ct^) method.

### Cell transfections

Short hairpin RNA (shRNA) against SOX2OT, SOX3 or TDGF-1 gene, as well as their non-targeting sequences were constructed in pGPU6/GFP/Neo vector (GenePharama, Shanghai, China). Full-length SOX3 or TDGF-1 gene were constructed in pIRES2-EGFP (GenScript, Piscataway, NJ, USA). MiR-194-5p agomir, miR-194-5p antagomir, miR-122 agomir, miR-122 antagomir and their respective negative control were synthesized (GenePharama, Shanghai, China). Cells were seeded in a 24-well plate (Corning, NY, USA), and Lipofectamine 3000 reagent and Opti-MEM I (Life Technologies, Waltham, MA) were used according to the manufacturer’s instructions to transfect cells with the plasmids when cells reached 70–80% confluence. G418 (Sigma-Aldrich, St Louis, MO, USA) was used to select the stable transfected cells. The transfection efficacy was analyzed with qRT-PCR or Western blotting. To evaluate the effect of SOX2OT on GSCs, cells were divided into three groups: control, sh-NC and sh-SOX2OT groups. To evaluate the effect of miR-194-5p on GSCs, cells were divided into five groups: control, agomir-194-5p-NC, agomir-194-5p, antagomir-194-5p-NC and antagomir-194-5p groups. To evaluate the effect of miR-122 on GSCs, cells were divided into five groups: control, agomir-122-NC, agomir-122, antagomir-122-NC and antagomir-122 groups. To determine whether SOX2OT-mediated regulation of miR-194-5p expression could affect the behaviors of GSCs, cells were divided into five groups: control, sh-NC + agomir-194-5p-NC, sh-SOX2OT + agomir-194-5p, sh-NC + antagomir-194-5p-NC and sh-SOX2OT + antagomir-194-5p groups. To determine whether SOX2OT-mediated regulation of miR-122 expression could affect the behaviors of GSCs, cells were divided into five groups: control, sh-NC + agomir-122-NC, sh-SOX2OT + agomir-122, sh-NC + antagomir-122-NC and sh-SOX2OT + antagomir-122 groups. To evaluate the effect of SOX3 on GSCs, cells were divided into five groups: control, SOX3(+)NC, SOX3(+), SOX3(−)NC and SOX3(−) groups. To determine whether SOX3 is involved in the miR-194-5p effect on the behaviors of GSCs, cells were divided into five groups: control, agomir-194-5p-NC + SOX3(+)NC, agomir-194-5p + SOX3(+)NC, agomir-194-5p-NC + SOX3(+) and agomir-194-5p + SOX3(+) groups. To determine whether SOX3 is involved in the of miR-122 effect on the behaviors of GSCs, cells were divided into five groups: control, agomir-122-NC + SOX3(+)NC, agomir-122 + SOX3(+)NC, agomir-122-NC + SOX3(+) and agomir-122 + SOX3(+) groups. To evaluate the effect of TDGF-1 on GSCs, cells were divided into five groups: control, TDGF-1(+)NC, TDGF-1(+), TDGF-1(−)NC and TDGF-1(−) groups.

### Cell proliferation assay

Cells were seeded in 96-well plates at a density of 2000 cells per well, and 20 μl of Cell Counting Kit-8 (Beyotime Institute of Biotechnology, Jiangsu, China) was added to each well after 48 h. Cells were incubated for 2 h at 37 °C and the absorbance was recorded at 450 nm.

### Cell migration and invasion assays

Cells were resuspended in 100 μl of serum-free medium at a density of 2 × 10^5^ cells/ml and seeded into the upper chamber (pre-coated with 50 ng/μl Matrigel solution (BD, Franklin Lakes, NJ, USA) for the cell invasion assay) with an 8 μm pore size polycarbonate membrane (Corning, NY, USA). Then, 600 μl of 10% FBS medium was placed in the lower chamber. After incubation at 37 °C for 48 h, the cells on the upper membrane surface were mechanically removed. Cells that migrated or invaded the lower surface of the membrane were fixed with methanol and glacial acetic acid at a ratio of 3:1 and stained with 20% Giemsa. Five random fields were chosen to count and take photos under a microscope.

### Quantization of apoptosis by flow cytometry

Cell apoptosis was assessed with Annexin V-FITC/PI staining (BD Biosciences). After being washed twice with PBS, cells were harvested in binding buffer at a concentration of 1 × 10^6^ cells/ml, and 5 μl of PI and 5 μl FITC were added to the cell suspension and incubated at room temperature in the dark for 15 min. Cell samples were analyzed via flow cytometry (FACScan, BD Biosciences), and apoptotic fractions were determined.

### Western blot analysis

Cells were lysed using RIPA (Beyotime Institute of Biotechnology) buffer on ice for 30 min and were centrifuged at 17,000×g for 45 min at 4 °C. The protein concentrations were analyzed by the BCA protein assay kit (Beyotime Institute of Biotechnology, Jiangsu, China). The samples were subjected to SDS-PAGE electrophoretically transferred to PVDF membranes. Membranes were blocked by Tween-Tris-buffered saline (TTBS) containing 5% non-fat milk for 2 h at room temperature and then incubated with primary antibodies as follows: SOX3 (1:1000, Santa Cruz Biotechnology), TDGF-1 (1:800, Abcam, UK), JAK-1, p-JAK-1, STAT3, (1:1000, Abcam, UK), p-STAT3 (1:1000, CST, EUGENE), and GAPDH (1:1000, Santa Cruz Biotechnology) overnight at 4 °C. Membranes were then washed three times with TTBS and incubated with horseradish peroxidase conjugated secondary antibody for 2 h at room temperature. The blots were visualized with enhanced chemiluminescence (ECL) kit (Santa Cruz Biotechnology) and scanned by ChemImager 5500 V2.03 software. The relative integrated density values (IDVs) were calculated using Fluor Chen 2.0 software based on GAPDH as an internal control.

### Reporter vectors construction and luciferase assays

The sequence of SOX2OT was amplified by PCR and cloned into pmirGLO Dual-luciferase miRNA Target Expression Vectors along with its mutant sequence of mir-194-5p (or mir-122) binding sites (GenePharama, Shanghai, China). HEK-293 T cells were seeded in a 96-well plate (Corning) and co-transfected with wild-type pmirGLO-SOX2OT (or SOX2OT mutant) reporter plasmid and agomir-194-5p (or agomir-122) or agomir-194-5p-NC (or agomir-122-NC), respectively. The luciferase activities were performed with the Dual-Lucifer Reporter Assay System (Promega, Madison, WI, USA) after 48 h according to the manufacturer’s instructions. The relative luciferase activity was calculated by normalizing to renilla luciferase activity. The 3′-UTR sequence of SOX3 and its mutant sequence of mir-194-5p (or mir-122) binding sites were cloned into pmirGLO Dual-luciferase miRNA Target Expression Vectors (GenePharama, Shanghai, China). The transfection procedure and calculating method of Lucifer’s activities were similarly as described above.

### Chromatin immunoprecipitation (ChIP) assay

ChIP assay was performed with Simple ChIP Enzymatic Chromatin IP Kit (Cell signaling Technology, Danvers, Massachusetts, USA) according to the manufacturer’s instructions. Cells were cross-linked with 1% formaldehyde for 10 min and then quenched with glycine. Cells were then collected in lysis buffer. 2% lysates were used as an input reference and other lysates were incubated with normal rabbit IgG or anti-SOX3 antibody with rotation. DNA crosslinks were reversed by NaCl and proteinase K and purified. DNA was amplified by PCR with following primers: the putative binding site of SOX3 in SOX2OT promoter using the primers 5′- TGCAGGAAGCAGGAGAATGG -3′ and 5′- CCGTTACGTTTTGCAAGCCA -3′, yielding a 199 bp product, control using the primers 5′- TCTTCCTAGGACAAAATCCCCC -3′ and 5′- GACAAAACGGGAAGCAGCATT -3′, yielding a 155 bp product; the putative binding site of SOX3 in TDGF-1 promoter using the primers 5′- GTCTTCCCCACACACACACA -3′ and 5′- TGTATGGGTCTCAAGGCATTC -3′, yielding a 186 bp product, control using the primers 5′- AGCGCCAAACTCCAGTCTAC -3′ and 5′- GACTGCAGAGGAAGCCAAGT -3′, yielding a 200 bp product.

### Tumor xenograft implantation in nude mice

For the in vivo study, the stably transfected cells were used. The mice were divided into five groups: control, sh-SOX2OT, miR-194-5p, miR-122 and sh-SOX2OT + miR-194-5p + miR-122 groups. Cells stably transfected with sh-SOX2OT were selected as described before. The pGCMV/EGFP/miR-194-5p plasmid and pGCMV/EGFP/miR-122 plasmid were transfected into cells respectively. After infection, the stable expressing cells of miR-194-5p and miR-122 were picked. Transfect pGCMV/EGFP/miR-194-5p plasmid and pGCMV/EGFP/miR-122 plasmid in sh-SOX2OT stable expressing cells to generate sh-SOX2OT + miR-194-5p + miR-122 stable expressing cell lines. Four weeks old athymic nude mice (BALB/c) were purchased from the Cancer Institute of the Chinese Academy of Medical Science. Experiments with mice were conducted strictly in accordance with a protocol approved by the Administrate Panel on Laboratory Animal Care of China Medical University.

Each nude mouse was subcutaneously injected with 3 × 10^5^ cells in the right flank area for subcutaneous implantation. Tumors were measured every five days and calculated according to the formula: volume (mm^3^) = length × width^2^/2. 45 days after injection, the mice were sacrificed and tumors were isolated. For orthotopic inoculations, the number of survived nude mice was registered and survival analysis was performed using Kaplan-Meier survival curve.

### Statistical analysis

Experimental date were presented as means ± standard deviation (SD). All differ-ences were analyzed by SPSS 18.0 statistical software with the Student’s t-test (two tailed) or one-way ANOVA. Differences were considered as statically significant when *P* < 0.05.

## Results

### Knockdown of SOX2OT inhibited proliferation, migration and invasion and promoted apoptosis in GSCs

**Fig. 1:**
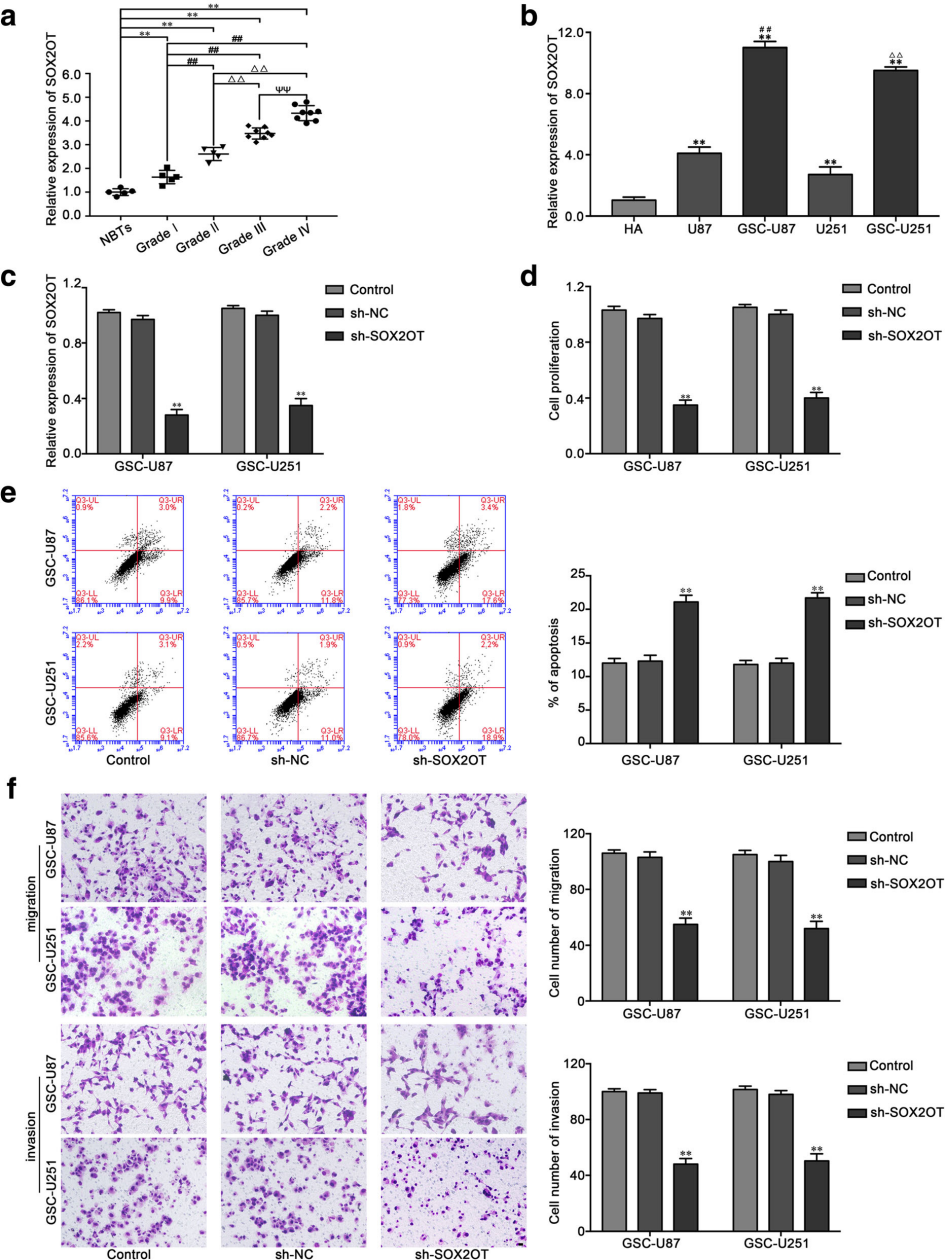
The SOX2OT expression and effects of SOX2OT in glioma. **a** The expression of SOX2OT in normal brain tissues (NBTs) and glioma tissues of different grades. Data are presented as the mean ± SD (NBTs (*n* = 5), Grade I(n = 5),Grade II(n = 5), Grade III (*n* = 8), Grade IV (n = 8)). ^**^ *P* < 0.01 vs. NBTs group; ^##^ *P* < 0.01 vs. Grade I group; ^△△^ *P* < 0.01 vs. Grade II group; ^ΨΨ^ *P* < 0.01 vs. Grade III group. **b** The expression of SOX2OT in human astrocytes (HA), glioblastoma cell lines (U87 and U251) and glioblastoma stem cells (GSC-U87, GSC-U251). Data are presented as the mean ± SD (*n* = 5, each group). ^**^ *P* < 0.01 vs. HA group; ^##^ *P* < 0.01 vs. U87 group; ^ΨΨ^ *P* < 0.01 vs. U251 group. **c** The expression of SOX2OT after cells transfection with sh-SOX2OT plasmids. **d** CCK-8 assay was used to measure the effect of SOX2OT on the proliferation of GSC-U87 and GSC-U251. **e** The apoptotic percentages of GSC-U87 and GSC-U251 were detected after SOX2OT knockdown. **f** Transwell assays were used to measure the effect of SOX2OT on cell migration and invasion of GSC-U87 and GSC-U251. Data represent as the mean ± SD (*n* = 5,each group). ^**^ *P* < 0.01 vs. sh-NC group. Scale bars represent 40 μm

The expression levels of SOX2OT in human glioma tissues, GBM cells and GSCs were evaluated by qRT-PCR. As shown in Fig. 1a, SOX2OT expression was significantly increased in glioma tissues of different grades compared with normal brain tissues (NBTs), and the expression was positively correlated with the tumor grade. Moreover, we also found that SOX2OT expression in human GBM cell lines and GSCs is higher than in HA cells, and was significantly higher in GSC-U87 and GSC-U251 cells than in U87 and U251 cells, respectively (Fig. 1b). We further analyzed the effect of SOX2OT knockdown on the proliferation, migration, invasion and apoptosis of GSC-U87 and GSC-U251 cells. The plasmids of 4 SOX2OT shRNAs were constructed, and cells were transfected with these shRNAs. The knockdown efficiency of 4 shRNAs were detected, and the most knockdown efficiency of SOX2OT shRNA (sh-SOX2OT-2) was selected for the subsequent experiments (Additional file 1: Figure S1). The knockdown efficiency in GSCs transfected with SOX2OT knockdown plasmids was shown in Fig. 1c. As shown in Fig. 1d, the proliferation of GSC-U87 and GSC-U251 cells was decreased in the sh-SOX2OT group compared with the sh-NC group. Flow cytometry analysis showed that SOX2OT knockdown promoted the apoptosis in GSC-U87 and GSC-U251 cells compared with the sh-NC group (Fig. 1e). As shown in Fig. 1f, the migration and invasion abilities in the sh-SOX2OT group were inhibited compared with those in the sh-NC group. The effects of SOX2OT knockdown in GSC-GBM were similar as it in GSC-U87 and GSC-U251 cells (Additional file 1: Figure S2). The above results indicated that SOX2OT functions as an oncogene in GSCs.

### MiR-194-5p and miR-122 functioned as tumor suppressors in GSCs

**Fig. 2:**
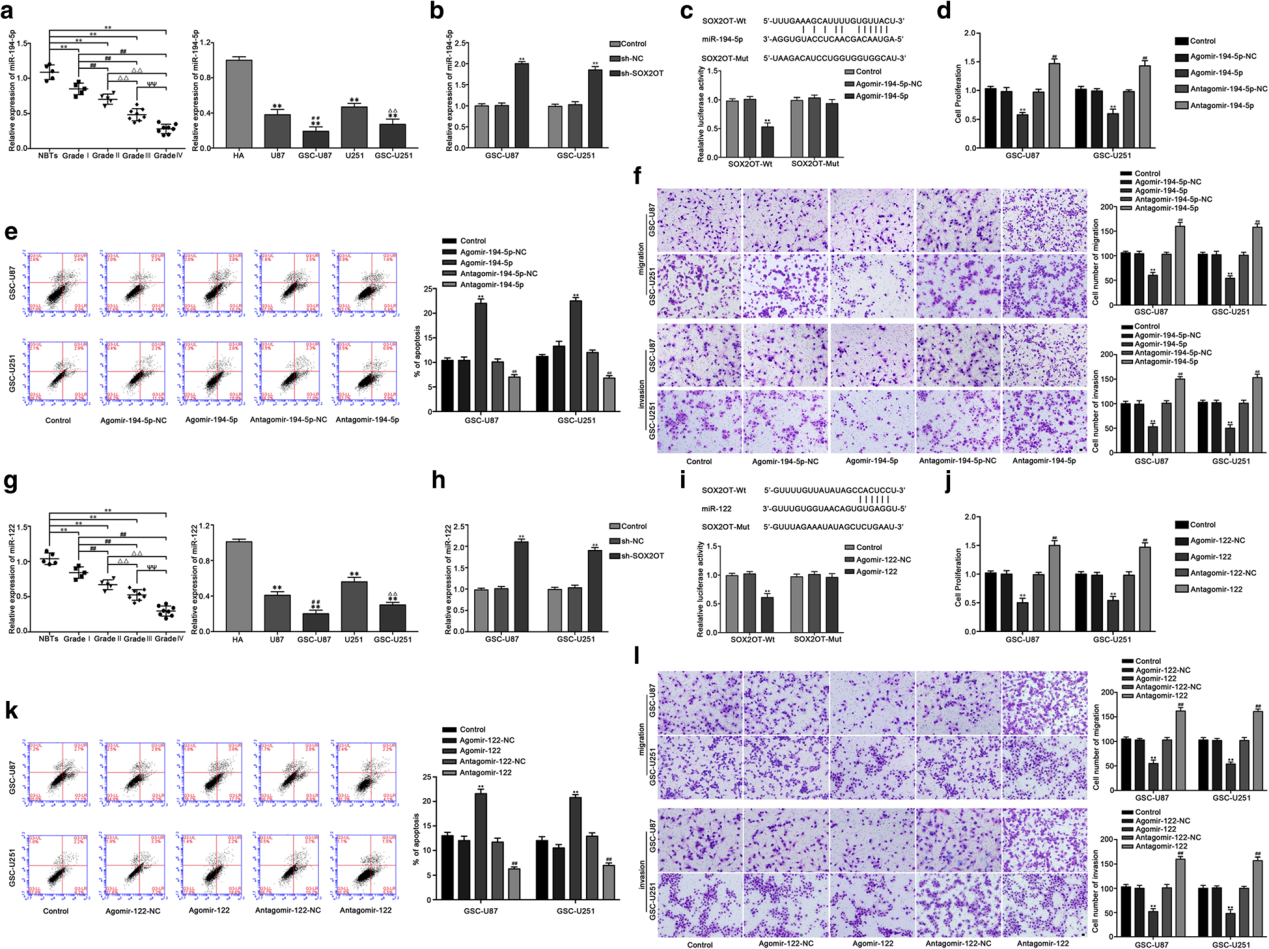
MiR-194-5p and miR-122 exerted tumor-suppressive functions in GSCs. **a** The expression of miR-194-5p in normal brain tissues (NBTs) and glioma tissues of different grades. Data are presented as the mean ± SD (NBTs (*n* = 5), Grade I (n = 5), Grade II (n = 5), Grade III (n = 8), Grade IV (n = 8)). ^**^ *P* < 0.01 vs. NBTs group; ^##^ *P* < 0.01 vs. Grade I group; ^△△^ *P* < 0.01 vs. Grade II group; ^ΨΨ^ *P* < 0.01 vs. Grade III group. The expression of miR-194-5p in human astrocytes (HA), glioblastoma cell lines (U87 and U251) and glioblastoma stem cells (GSC-U87, GSC-U251). Data are presented as the mean ± SD (n = 5,each group). ^**^ *P* < 0.01 vs. HA group; ^##^ *P* < 0.01 vs. U87 group; ^△△^ *P* < 0.01 vs. U251 group. **b** The expression of miR-194-5p after SOX2OT knockdown in GSC-U87 and GSC-U251 cells. ^**^ *P* < 0.01 vs. sh-NC group. **c** The predicted miR-194-5p binding site in the SOX2OT sequence (SOX2OT-Wt) and the designed mutant sequence of miR-194-5p binding site (SOX2OT-Mut) are indicated. Relative luciferase activity was conducted after cells were transfected with SOX2OT-Wt or SOX2OT-Mut. Data were presented as the mean ± SD (n = 5, each group). ^**^ *P* < 0.01 vs. SOX2OT-Wt + Agomir-194-5p-NC. **d** CCK-8 assay was used to measure the effect of miR-194-5p on the proliferation of GSC-U87 and GSC-U251 cells. **e** The apoptotic percentages of GSC-U87 and GSC-U251 cells were detected after miR-194-5p over-expression or inhibition. **f** Transwell assays was used to measure the effect of miR-194-5p on the migration and invasion of GSC-U87 and GSC-U251 cells. Scale bars represent 40 μm. Data are presented as the mean ± SD (*n* = 5, each group).^**^ *P* < 0.01 vs. Agomir-194-5p-NC group; ^##^ *P* < 0.01 vs. Antagomir-194-5p-NC group. **g** The expression of miR-122 in normal brain tissues(NBTs) and glioma tissues of different grades. Data are presented as the mean ± SD (NBTs (n = 5), Grade I (n = 5), Grade II(n = 5), Grade III (n = 8), Grade IV (n = 8)). ^**^ *P* < 0.01 vs. NBTs group; ^##^ *P* < 0.01 vs. Grade I group; ^△△^ *P* < 0.01 vs. Grade II group; ^ΨΨ^ *P* < 0.01 vs. Grade III group. The expression of miR-122 in HA cells, glioblastoma cell lines(U87 and U251) and glioblastoma stem cells (GSC-U87, GSC-U251). Data are presented as the mean ± SD (n = 5,each group). ^**^ *P* < 0.01 vs. HA group; ^##^ *P* < 0.01 vs. U87 group; ^△△^ *P* < 0.01 vs. U251 group. **h** The expression of miR-122 with SOX2OT knockdown in GSC-U87 and GSC-U251 cells. ^**^ *P* < 0.01 vs. sh-NC group. **i** The predicted miR-122 binding sites in the SOX2OT sequence (SOX2OT-Wt) and the designed mutant sequence of miR-122 binding site (SOX2OT-Mut) are indicated. Relative luciferase activity was conducted after cells were transfected with SOX2OT-Wt or SOX2OT-Mut. Data were presented as the mean ± SD (n = 5, each group). ^**^ *P* < 0.01 vs. SOX2OT-Wt + Agomir-122-NC. **j** CCK-8 assay was used to measure the effect of miR-122 on the proliferation of GSC-U87 and GSC-U251 cells. **k** The apoptotic percentages of GSC-U87 and GSC-U251 cells were detected after miR-122 over-expression or inhibition. **l** Transwell assays were used to measure the effect of miR-122 on the migration and invasion of GSC-U87 and GSC-U251 cells. Scale bars represent 40 μm. Data are presented as the mean ± SD (*n* = 5, each group). ^**^ *P* < 0.01 vs. Agomir-122-NC group; ^##^ *P* < 0.01 vs. Antagomir-122-NC group

The expression levels of miR-194-5p in human glioma tissues, GBM cells and GSCs were measured by qRT-PCR. As shown in Fig. 2a, miR-194-5p were significantly decreased in glioma tissues of different grades, compared with normal brain tissues (NBTs), and the expression was negatively correlated with tumor grade. In addition, the expression of miR-194-5p in GBMs and GSCs was decreased compared with that in HA cells, and reduced in GSC-U87 and GSC-U251 cells compared with U87 and U251 cells, respectively. Emerging evidences has shown that lncRNA might be a molecular sponge or a competing endogenous RNA (ceRNA) that modulates miRNA. According to the bioinformatics database (Starbase v2.0), we proposed that SOX2OT might harbor miR-194-5p binding sites. The expression level of miR-194-5p was increased in the sh-SOX2OT group compared with the sh-NC group (Fig. 2b). To elucidate the molecular mechanisms, luciferase reporter assay was conducted. The results showed that the luciferase activities in the SOX2OT-Wt + Agomir-194-5p group were inhibited (Fig. 2c), suggesting that SOX2OT targeted miR-194-5p and regulated its expression.

To investigate the miR-194-5p effect on GSCs, we next detected cell proliferation, migration, invasion and apoptosis of GSCs after miR-194-5p over-expression or inhibition. As shown in Fig. 2d, the proliferation of GSC-U87 and GSC-U251 cells was decreased in the Agomir-194-5p group compared with the Agomir-194-5p-NC group, whereas the proliferation of GSC-U87 and GSC-U251 cells was increased in the Antagomir-194-5p group compared with the Antagomir-194-5p-NC group. Flow cytometry analysis showed that the apoptosis of GSC-U87 and GSC-U251 cells was increased in the Agomir-194-5p group compared with the Agomir-194-5p-NC group, whereas the apoptosis of GSC-U87 and GSC-U251 cells was decreased in the Antagomir-194-5p group compared with the Antagomir-194-5p-NC group (Fig. 2e). As shown in Fig. 2f, the migration and invasion abilities of GSC-U87 and GSC-U251 cells were decreased in the Agomir-194-5p group compared with the Agomir-194-5p-NC group, whereas the migration and invasion abilities of GSC-U87 and GSC-U251 cells were increased in the Antagomir-194-5p group compared with the Antagomir-194-5p-NC group. Similar results were also observed when detecting the effect of miR-122 on the proliferation, migration, invasion and apoptosis of GSCs (Fig. 2g–l). The effects of mir-194-5p and miR-122 in GSC-GBM were similar as it in GSC-U87 and GSC-U251 cells (Additional file 1: Figure S3). These results demonstrated that miR-194-5p and miR-122 exerted the tumor-suppressive role in GSCs.

### MiR-194-5p and miR-122 mediated the tumor-suppressive effects of SOX2OT knockdown on GSCs

**Fig. 3:**
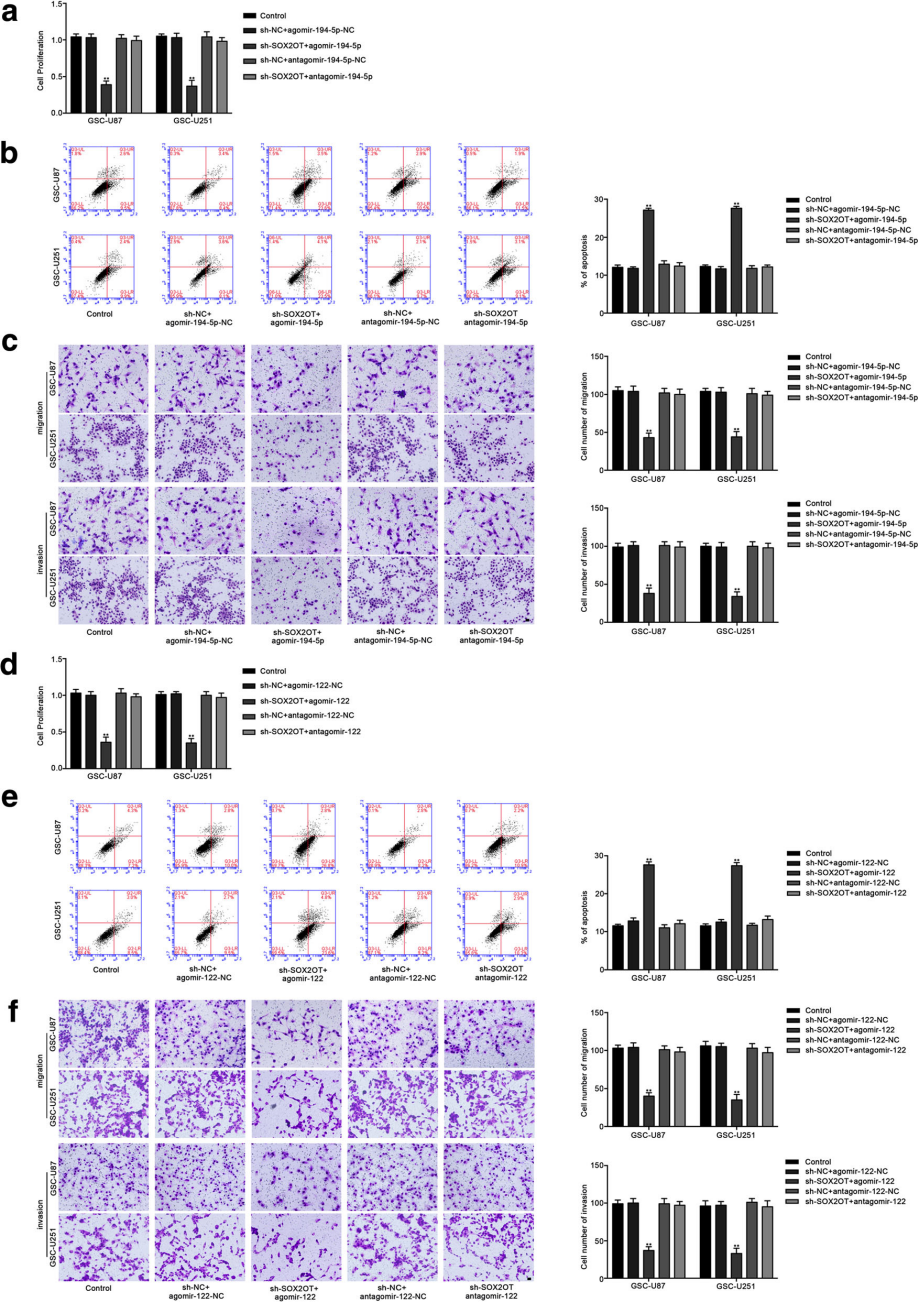
MiR-194-5p and miR-122 mediated the tumor-suppressive effects of SOX2OT knockdown on GSCs. **a** CCK-8 assay was used to measure the effect of SOX2OT and miR-194-5p on the proliferation of GSC-U87 and GSC-U251 cells. **b** Flow cytometry analysis to evaluate the effect of SOX2OT and miR-194-5p on the apoptosis of GSC-U87 and GSC-U251 cells. **c** Transwell assays was used to measure the effect of SOX2OT and miR-194-5p on the migration and invasion of GSC-U87 and GSC-U251 cells. Data are presented as the mean ± SD (*n* = 5, each group).^**^ *P* < 0.01 vs. sh-NC + agomir-194-5p-NC. **d** CCK-8 assay was used to measure the effect of SOX2OT and miR-122 on the proliferation of GSC-U87 and GSC-U251 cells. **e** Flow cytometry analysis to evaluate the effect of SOX2OT and miR-122 on the apoptosis of GSC-U87 and GSC-U251 cells. **f** Transwell assays was used to measure the effect of SOX2OT and miR-122 on the migration and invasion of GSC-U87 and GSC-U251 cells. Scale bars represent 40 μm. Data are presented as the mean ± SD (*n* = 5,each group) . ^**^ *P* < 0.01 vs. sh-NC + agomir-122-NC

To determine whether the tumor-suppressive effects of SOX2OT knockdown were mediated by miR-194-5p or miR-122, the stable sh-SOX2OT cells were transfected with miR-194-5p or miR-122 agomir and antagomir. Stable co-transfection of sh-SOX2OT cells with agomir-194-5p had the strongest inhibitory effect on cell proliferation, migration and invasion, and promoted the apoptosis of GSCs. Moreover, transfection with antagomir-miR-194-5p rescued the inhibitory effect of sh-SOX2OT on cell proliferation, migration and invasion, and rescued the increased apoptosis induced in the sh-SOX2OT group (Fig. 3a–c). Similar results were observed with miR-122 (Fig. 3d–f). Based on the above results, we confirmed that miR-194-5p and miR-122 mediate the tumor-suppressive effects of SOX2OT knockdown in GSCs, and knockdown of miR-194-5p or miR-122 respectively reversed the effects induced by SOX2OT knockdown in GSCs.

### SOX3 acted as an oncogene and transcriptionally activated the expression of SOX2OT and TDGF-1 in GSCs

**Fig. 4:**
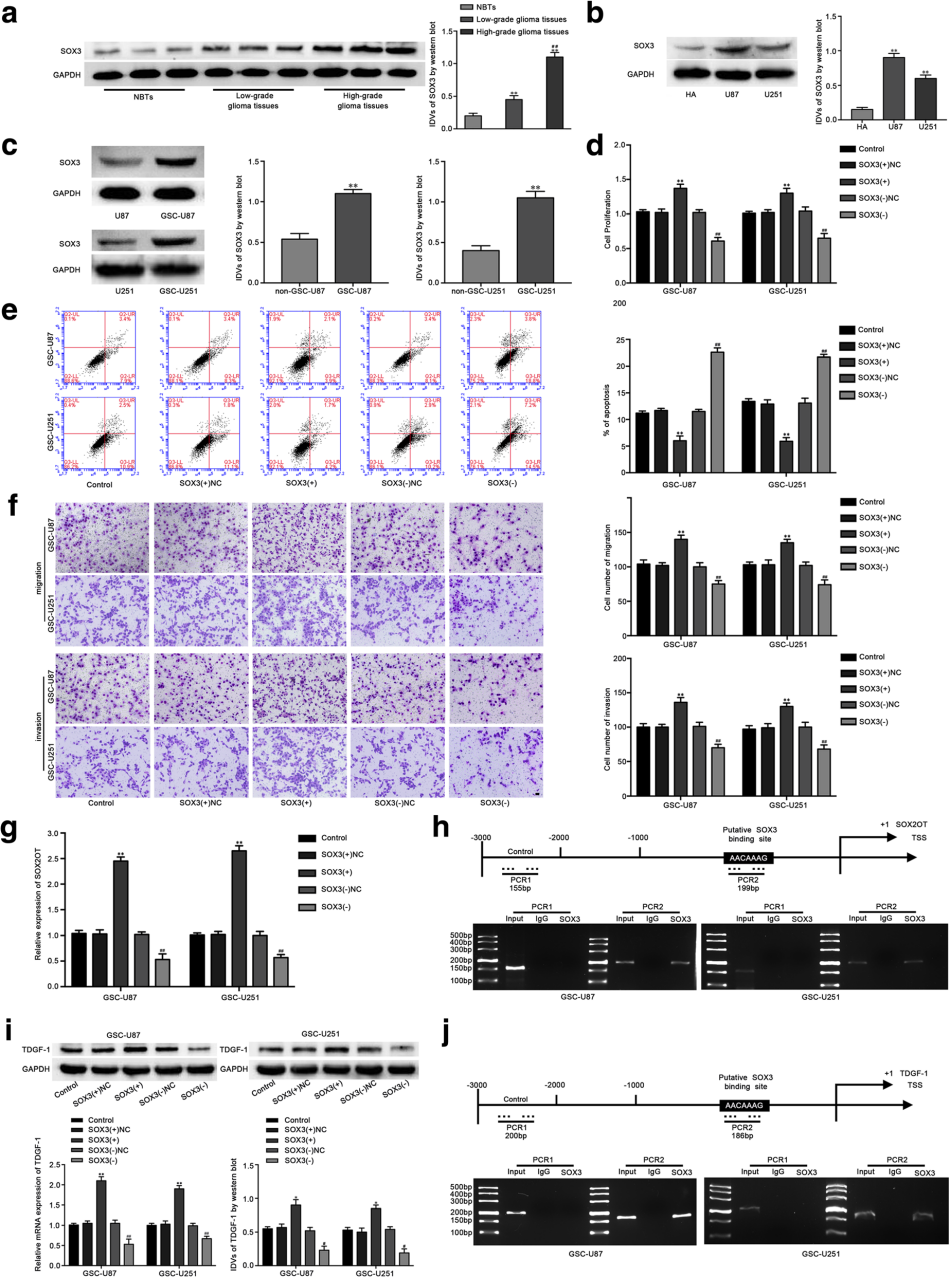
SOX3 endogenous expression and its effect on the proliferation, migration, invasion and apoptosis in GSCs, as well as the expression of SOX2OT and TDGF-1. **a** The SOX3 protein expression levels in normal brain tissues (NBTs), low-grade glioma tissues (WHO I-II) and high-grade glioma tissues (WHO III-IV) are shown. Data are presented as the mean ± SD (*n* = 3,each group). ^**^ *P* < 0.01 vs. NBTs group; ^##^ *P* < 0.01 vs. low-grade glioma tissues group. **b**The expression level of SOX3 in human astrocytes (HA) and glioblastoma cell lines (U87 and U251). **c** The expression of SOX3 in glioblastoma cell lines (U87 and U251) and glioblastoma stem cells (GSC-U87, GSC-U251). **d** CCK-8 assay was used to measure the effect of SOX3 on the proliferation of GSC-U87 and GSC-U251 cells. **e** The apoptotic percentages of GSC-U87 and GSC-U251 were detected after SOX3 over-expression or knockdown. **f** Transwell assays was used to measure the effect of SOX3 on cell migration and invasion of GSC-U87 and GSC-U251 cells. Scale bars represent 40 μm. Data are presented as the mean ± SD (*n* = 5, each group). ^**^ *P* < 0.01 vs. SOX3(+)NC group; ^##^ *P* < 0.01 vs. SOX3(−)NC group. **g** Relative expression of SOX2OT with the over-expressing or silencing SOX3. **h** SOX3 bound to the promoter of SOX2OT in GSC-U87 and GSC-U251 cells. Schematic representation of the human SOX2OT promoter region 3000 bp upstream of the transcription start site (TSS), which was designated as +1. Putative SOX3 binding site was indicated. PCR was conducted with the resulting precipitated DNA. **i** Weatern blot assay was used to detect the expression of TDGF-1 after SOX3 over-expression or knockdown. **j** SOX3 bound to the promoter of TDGF-1 in GSC-U87 and GSC-U251 cells. Schematic representation of the human TDGF-1 promoter region 3000 bp upstream of the transcription start site (TSS), which was designated as +1. Putative SOX3 binding site was indicated. PCR was conducted with the resulting precipitated DNA

Western blotting was performed to analyze the expression of SOX3 in human glioma tissues, GBM cells and GSCs. As shown in Fig. 4a, SOX3 expression was obviously up-regulated in glioma tissues compared with NBTs and up-regulated in high-grade glioma tissues compared with low-grade glioma tissues. In addition, the expression of SOX3 in U87 and U251 glioma cell lines was higher than that in HA cells, but lower than that in GSC-U87 and GSC-U251 cells (Fig. 4b, c). To explore the function of SOX3 in GSCs, the biological behaviors of GSCs were detected. As shown in Fig. 4d, cell proliferation was increased in the SOX3(+) group, and decreased in the SOX3(−) group (Fig. 4d). Flow cytometry revealed that over-expression of SOX3 suppressed the cell apoptosis, whereas knockdown of SOX3 enhanced the apoptosis of GSC-U87 and GSC-U251 cells (Fig. 4e). As shown in Fig. 4f, the number of migration and invasion of GSC-U87 and GSC-U251 cells was increased in the SOX3(+) group compared with the SOX3(+)NC group, and decreased in the SOX3(−) group compared with the SOX3(−)NC group. The effects of SOX3 in GSC-GBM were similar as it in GSC-U87 and GSC-U251 cells (Additional file 1: Figure S4A-D). These results indicated that the SOX3 functions as an oncogene in GSCs.

**Fig. 5:**
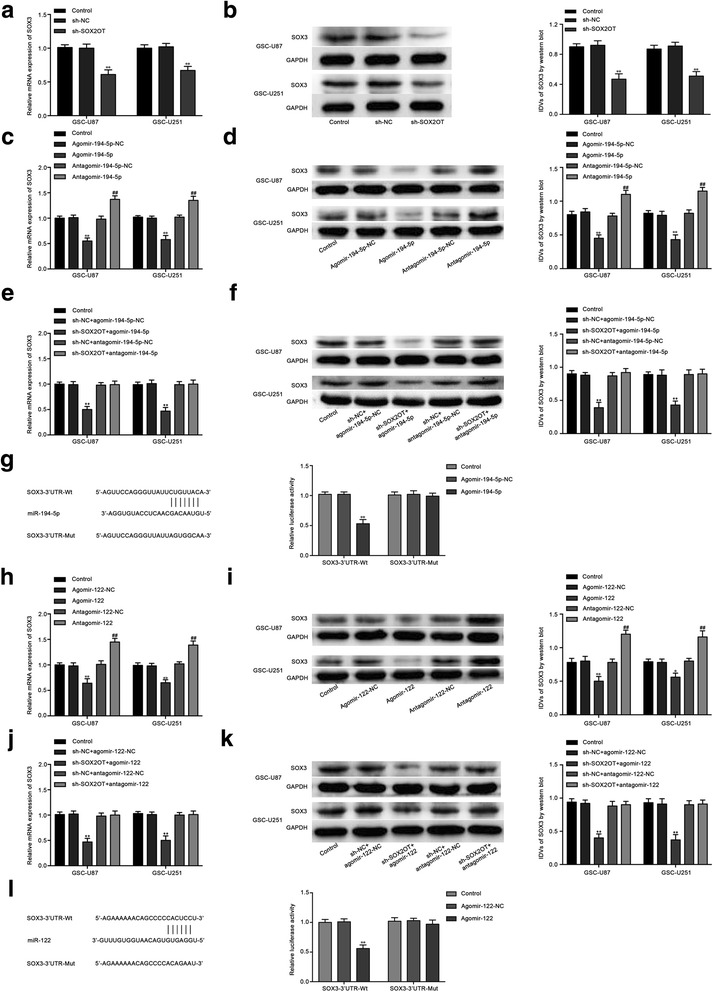
The SOX3 expression regulated by SOX2OT, miR-194-5p and miR-122. **a** Real-time PCR and (**b**) Weatern blot assay were used to detect the SOX3 expression after SOX2OT knockdown. **c** Real-time PCR and (**d**) Weatern blot assay were used to detect the SOX3 expression after miR-194-5p over-expression or knockdown. **e** Real-time PCR and (**f**) Weatern blot assay were used to detect the SOX3 expression regulated by SOX2OT and miR-194-5p. **g** The predicted miR-194-5p binding sites in the 3’UTR region of SOX3 (SOX3–3’UTR-Wt) and the designed mutant sequence (SOX3–3’UTR-Mut) are indicated. Relative luciferase activity was conducted after cells were transfected with SOX3–3’UTR-Wt or SOX3–3’UTR-Mut. Data were presented as the mean ± SD (*n* = 5,each group).^**^ *P* < 0.01 vs. SOX3–3’UTR-Wt + Agomir-194-5p-NC group. **h** Real-time PCR and (**i**) Weatern blot assay were used to detect the SOX3 expression after miR-122 over-expression or knockdown. **j** Real-time PCR and (**k**)Weatern blot assay were used to detect the SOX3 expression regulated by SOX2OT and miR-122. **l** The predicted miR-122 binding sites in the 3’UTR region of SOX3 (SOX3–3’UTR-Wt) and the designed mutant sequence (SOX3–3’UTR-Mut) are indicated. Data were presented as the mean ± SD (n = 5,each group). ^**^ *P* < 0.01 vs. SOX3–3’UTR-Wt + Agomir-122-NC group

We inspected the promoter sequence of SOX2OT, and one putative SOX3 binding site was found. Thus, SOX2OT expression was detected after SOX3 over-expression or knockdown. As shown in Fig. 4g, the SOX2OT expression in GSC-U87 and GSC-U251 cells was increased in the SOX3(+) group compared with the SOX3(+)NC group, while the expression was down-regulated in the SOX3(−) group compared with the SOX3(−)NC group. Additionally, chromatin immunoprecipitation (ChIP) assays were used to confirm the interaction between SOX2OT and the putative binding site of SOX3. As a negative control, PCR was used to amplify the region 1000 bp upstream of the putative SOX3 binding site that was not predicted to associate with SOX3. As shown in Fig. 5h, there was an association between SOX2OT and the putative binding site of SOX3.

TDGF-1 was predicted as a downstream target of SOX3 by the bioinformatic database (JASPAR). We next determined if SOX3 regulates TDGF-1 expression using Western blot assay. As shown in Fig. 4i, over-expression of SOX3 increased the expression level of TDGF-1, and SOX3 knockdown decreased TDGF-1 expression. ChIP assays were performed to explore the interaction between TDGF-1 and the putative binding site of SOX3. As a negative control, PCR was used to amplify the region 1000 bp upstream of the putative SOX3 binding site that was not predicted to associate with SOX3. As shown in Fig. 4j, there was an interaction between TDGF-1 and the putative binding site of SOX3. The above results revealed that SOX3 promoted the expression of SOX2OT and TDGF-1 by binding to their promoters in GSC-U87 and GSC-U251 cells.

### Knockdown of SOX2OT decreased SOX3 expression by up-regulating miR-194-5p and miR-122

The mRNA and protein expression levels of SOX3 were analyzed in stable sh-SOX2OT GSC-U87 and GSC-U251 cells. As shown in Fig. 5a and b, the mRNA and protein expression levels of SOX3 were significantly down-regulated in the sh-SOX2OT group compared with the sh-NC group. Nevertheless, mRNA and protein expression was decreased in the agomir-194-5p group, and increased in the antagomir-194-5p group compared with their respective NC groups (Fig. 5c, d). Moreover, co-transfection of the stable sh-SOX2OT cells with agomir-194-5p had the strongest inhibitory effect on the mRNA and protein expression of SOX3. Our results also showed that the mRNA and protein expression levels of SOX3 that should be reduced by SOX2OT knockdown were restored by antagomir-miR-194-5p (Fig. 5e, f). These results indicated that SOX2OT knockdown inhibited SOX3 expression by increasing miR-194-5p expression. Based on information obtained from a bioinformatic database (TargetScan), SOX3 might be a target of miR-194-5p. Luciferase reporter assays were used to confirm the existence of a putative binding site in the 3’UTR of SOX3. In the SOX3–3’UTR-Wt group, the luciferase activity of cells cotransfected with SOX3–3’UTR-Wt and agomir-194-5p was inhibited, while no change was observed in their NC group. In the SOX3–3’UTR-Mut group, the luciferase activity remained unchanged (Fig. 5g). The results confirmed our prediction that SOX3 is a direct target of miR-194-5p. In addition, similar results were also observed with miR-122 (Fig. 5h-l). The above results suggested that miR-194-5p and miR-122 reduced SOX3 expression by targeting its 3′-UTR and mediated the tumor-suppressive effect of SOX2OT knockdown.

### SOX3 mediated the tumor-suppressive effects of miR-194-5p and miR-122 in GSCs

**Fig. 6:**
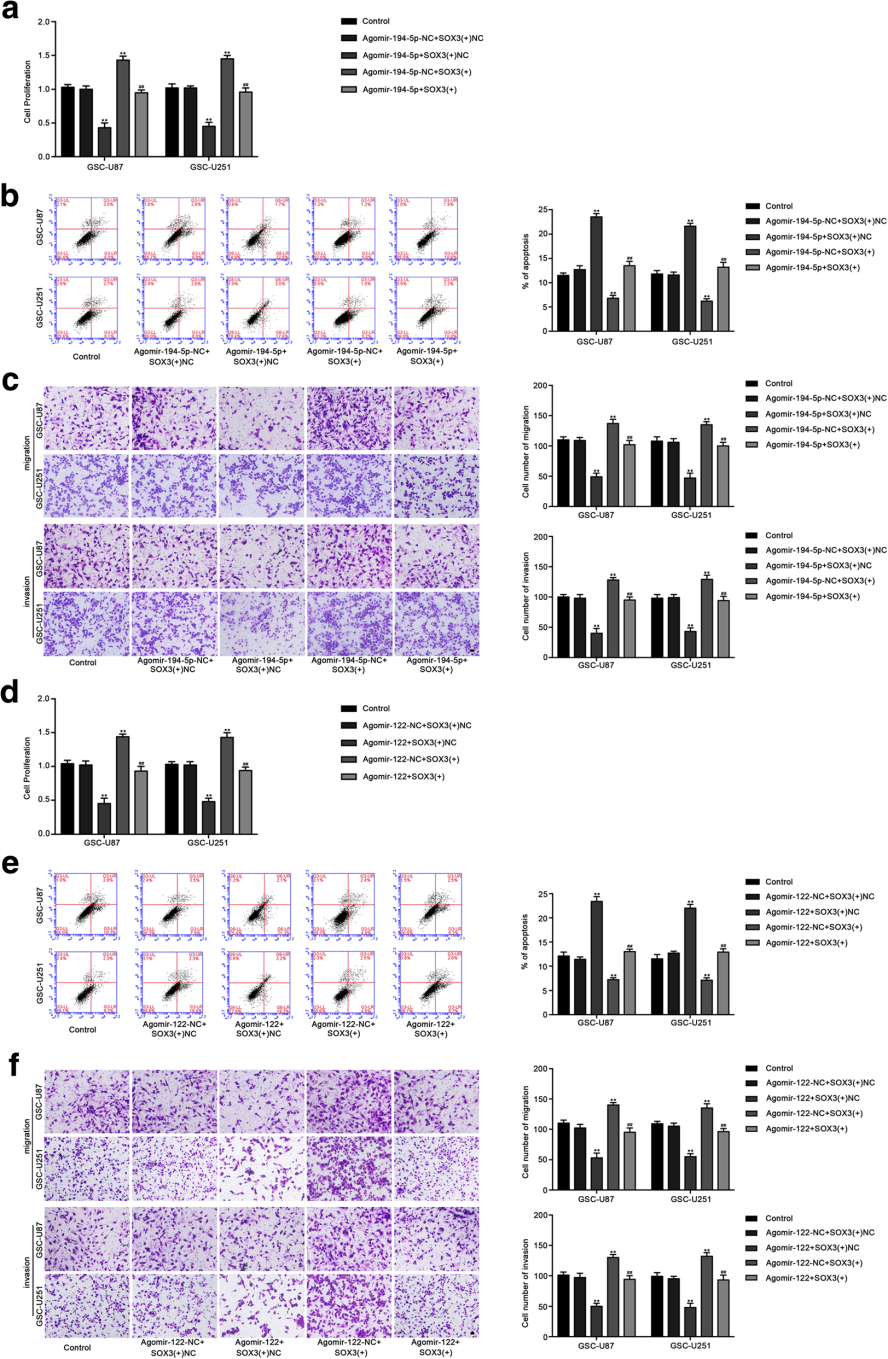
SOX3 mediated tumor-suppressive effects of miR-194-5p and miR-122. **a** CCK8 assay to evaluate the effect of miR-194-5p and SOX3 on cell proliferation of GSC-U87 and GSC-U251 cells. **b** Flow cytometry analysis to evaluate the effect of miR-194-5p and SOX3 on cell apoptosis of GSC-U87 and GSC-U251 cells. **c** Transwell assay to evaluate the effect of miR-194-5p and SOX3 on the cell migration and invasion of GSC-U87 and GSC-U251 cells. Data are presented as the mean ± SD (n = 5, each group). Scale bars represent 40 μm. ^**^ *P* < 0.01 vs. AgomiR-194-5p-NC + SOX3(+)NC group, ^##^ *P* < 0.01 vs. AgomiR-194-5p + SOX3(+)NC group. **d** CCK8 assay to evaluate the effect of miR-122 and SOX3 on cell proliferation of GSC-U87 and GSC-U251 cells. **e** Flow cytometry analysis to evaluate the effect of miR-122 and SOX3 on cell apoptosis of GSC-U87 and GSC-U251 cells. **f** Transwell assay to evaluate the effect of miR-122 and SOX3 on the cell migration and invasion of GSC-U87 and GSC-U251 cells. Data are presented as the mean ± SD (n = 5, each group). Scale bars represent 40 μm. ^**^ *P* < 0.01 vs. AgomiR-122-NC + SOX3(+)NC group, ^##^ *P* < 0.01 vs. AgomiR-122 + SOX3(+)NC group

To uncover whether SOX3 could reverse the tumor-suppressive effects of miR-194-5p and miR-122 in GSCs, cells were cotransfected with miR-194-5p or miR-122 and SOX3, and cell proliferation, migration, invasion and apoptosis were assessed. The mRNA and protein expression of SOX3 after cells cotransfection of miR-194-5p or miR-122 with SOX3 were shown in Additional file 1: Figure S5. Compared with the agomir-194-5p-NC + SOX3(+)NC group, the proliferation, migration and invasion of GSCs were reduced in the agomir-194-5p + SOX3(+)NC group and agomir-122 + SOX3(+)NC group, but increased in the agomir-194-5p-NC + SOX3(+) group and agomir-122-NC + SOX3(+) group. In addition, in the agomir-194-5p + SOX3(+) group and agomir-122 + SOX3(+)group, SOX3 rescued the inhibitory effect of agomir-194-5p + SOX3(+)NC and agomir-122 + SOX3(+)NC on the proliferation, migration and invasion of GSCs. (Fig. 6a–f). Moreover, cell apoptosis was increased in the agomir-194-5p + SOX3(+)NC group and agomir-122 + SOX3(+)NC group, and decreased in the agomir-194-5p-NC + SOX3(+) group and agomir-122-NC + SOX3(+) group. Similarly, overexpression of SOX3 hindered the increase in the apoptosis ratio caused by agomir-194-5p + SOX3(+)NC and agomir-122 + SOX3(+)NC (Fig. 6b, e). These results suggested that miR-194-5p and miR-122 suppressed the malignant behaviors of GSCs by down-regulating SOX3.

### TDGF-1 acted as an oncogene by activating the JAK/STAT signaling pathway, and miR-194-5p and miR-122 reduced TDGF-1 expression by down-regulating SOX3 expression in GSCs

**Fig. 7:**
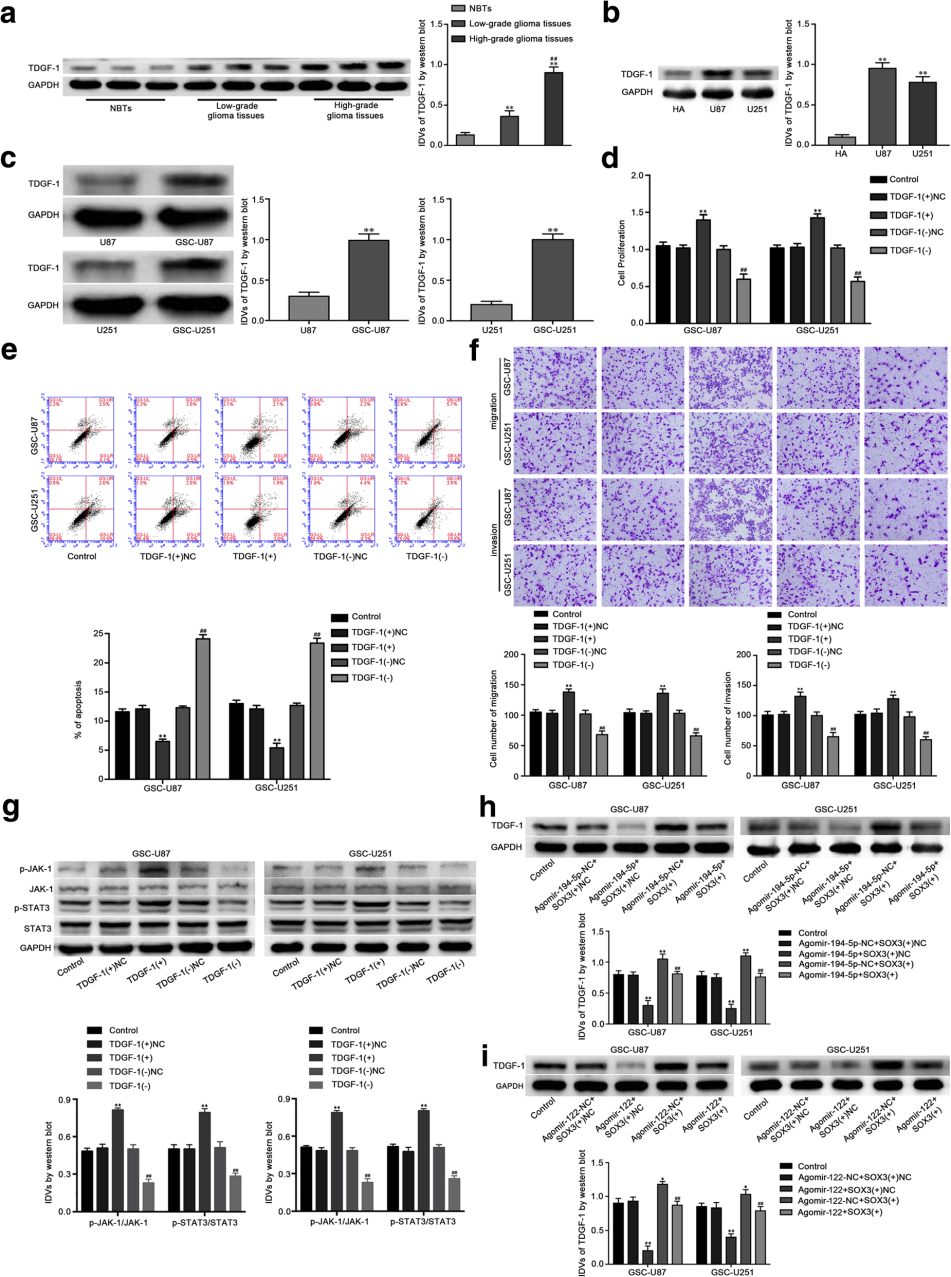
TDGF-1 endogenous expression and effect on proliferation, migration, invasion and apoptosis of GSCs. **a** TDGF-1 protein expression levels in normal brain tissues (NBTs), low-grade glioma tissues (WHO I-II) and high-grade glioma tissues (WHO III-IV) are shown. Data are presented as the mean ± SD (*n* = 3,each group). **b** The expression of TDGF-1 in human astrocytes (HA) and glioblastoma cell lines (U87 and U251). **c** The expression of TDGF-1 in glioblastoma cell lines (U87 and U251) and glioblastoma stem cells (GSC-U87, GSC-U251). **d** CCK-8 assay was used to measure the effect of TDGF-1 on the proliferation of GSC-U87 and GSC-U251 cells. **e** The apoptotic percentages of GSC-U87 and GSC-U251 were detected after TDGF-1 over-expression or knockdown. **f** Transwell assays were used to measure the effect of TDGF-1 on cell migration and invasion of GSC-U87 and GSC-U251 cells. Data are presented as the mean ± SD (n = 5, each group). ^**^ *P* < 0.01 vs. TDGF-1(+)NC, ^##^ *P* < 0.01 vs. TDGF-1(−)NC. **g** Western blot assay of the p-JAK-1/JAK-1 and p-STAT3/STAT3 expression regulated by TDGF-1. Data are presented as the mean ± SD (n = 5, each group). ^**^ *P* < 0.01 vs. TDGF-1(+)NC, ^##^ *P* < 0.01 vs. TDGF-1(−)NC. **h** Weatern blot assay were used to detect the TDGF-1 expression regulated by miR-194-5p and SOX3. Data are presented as the mean ± SD (n = 5, each group). ^**^ *P* < 0.01 vs. AgomiR-194-5p-NC + SOX3(+)NC group, ^##^ *P* < 0.01 vs. AgomiR-194-5p + SOX3(+)NC group. **i** Weatern blot assay were used to detect the TDGF-1 expression regulated by miR-122 and SOX3. Data are presented as the mean ± SD (n = 5, each group). ^**^ *P* < 0.01 vs. AgomiR-122-NC + SOX3(+)NC group, ^##^ *P* < 0.01 vs. AgomiR-122 + SOX3(+)NC group

TDGF-1 expression in human glioma tissues, GBMs and GSCs was detected. The expression of TDGF-1 in glioma tissues was significantly higher than that in NBTs, and upregulated in high-grade glioma tissues compared with low-grade glioma tissues (Fig. 7a). The expression level of TDGF-1 in GBM cells was higher than in HA cells (Fig. 7b). In addition, the expression level of TDGF-1 in GSC-U87 and GSC-U251 cells was increased compared with that in U87 and U251 cells, respectively (Fig. 7c). To further evaluate the impact of TDGF-1 on GSCs, cell proliferation, apoptosis, migration and invasion was detected after TDGF-1 over-expression or knockdown. As shown in Fig. 7d, over-expression of TDGF-1 elevated cell proliferation, while TDGF-1 inhibition reduced GSCs proliferation. Cell apoptosis in the TDGF-1(+) group was inhibited, and promoted in the TDGF-1(−) group compared with their respective NC group (Fig. 7e). Transwell assays showed that GSC-U87 and GSC-U251 cells in the TDGF-1(+) group had heightened migration and invasion ability, and weakened ability in the TDGF-1(−) group compared with their NC group (Fig. 7f). The effects of TDGF-1 in GSC-GBM were similar as it in GSC-U87 and GSC-U251 cells (Additional file 1: Figure S4E-H). These data suggested that TDGF-1 functions as an oncogene in GSCs.

The results of a previous study confirmed that STAT3 promoted glioma progression [44]. To further examine the molecular mechanism of the TDGF-1 oncogenic functions, JAK/STAT signaling pathway activity was detected by Western blot. As shown in Fig. 7g, over-expression of TDGF-1 activated the JAK/STAT signaling pathway by increasing the phosphorylation levels of JAK-1 and STAT3. Meanwhile, the expression levels of p-JAK-1 and p-STAT3 were decreased in the TDGF-1(−) group. However, the non-phosphorylated JAK-1 and STAT3 levels remained unchanged. These results demonstrated that TDGF-1 activated the JAK/STAT pathway and played an oncogenic role in GSCs.

We confirmed that TDGF-1 is a downstream target of SOX3, as shown in Fig. 4. To further detect whether miR-194-5p and miR-122 reduced TDGF-1 expression by down-regulating SOX3 expression, we detected TDGF-1 protein expression via western blotting. Compared with the agomir-194-5p-NC + SOX3(+)NC group or agomir-122-NC + SOX3(+)NC group, TDGF-1 expression was decreased in the agomir-194-5p + SOX3(+)NC group and agomir-122 + SOX3(+)NC group, and increased in the agomir-194-5p-NC + SOX3(+) group and agomir-122-NC + SOX3(+) group. In addition, SOX3 rescued the inhibitory effect of agomir-194-5p + SOX3(+)NC and agomir-122 + SOX3(+)NC on TDGF-1 expression in GSCs (Fig. 7h). These results revealed that miR-194-5p and miR-122 inhibited TDGF-1 expression by reducing SOX3.

### Knockdown of SOX2OT combined with over-expression of miR-194-5p and miR-122 suppressed tumor growth and induced the longest survival time in nude mice

**Fig. 8:**
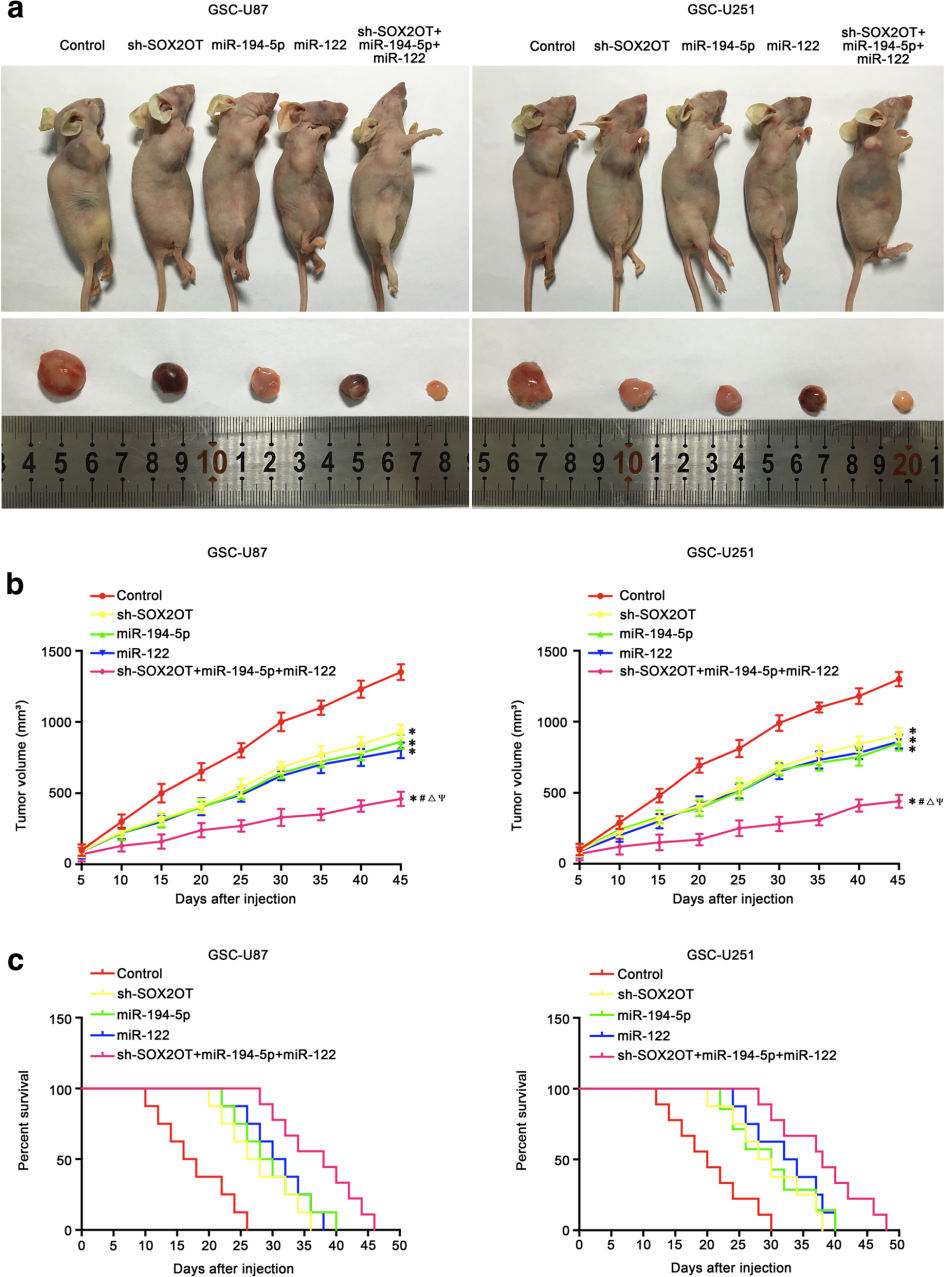
Tumor xenograft studies. **a** The nude mice carrying tumors from respective groups were shown. The sample tumors from respective group were shown. **b** Tumor growth curves were shown. Tumor volume was calculated every 5 days after injection, and the tumor was taken after 45 days. **c** Survival curves from representive nude mice injected into the right striatum were shown (*n* = 8, each group)

An in vivo tumor model was used to further determine the functions of SOX2OT, miR-194-5p and miR-122. As shown in Fig. 8a and b, SOX2OT inhibition, miR-194-5p over-expression, miR-122 over-expression and SOX2OT inhibition combined with over-expression of both miR-194-5p and miR-122 produced smaller tumors compared with the control group. Additionally, SOX2OT inhibition combined with over-expression of both miR-194-5p and miR-122 resulted in the smallest tumor size among all the groups. As shown in Fig. 8c, the survival analysis indicated that mice in the sh-SOX2OT, miR-194-5p, miR-122 and sh-SOX2OT + miR-194-5p + miR-122 groups exhibited longer survival time than the control mice, and mice in the sh-SOX2OT + miR-194-5p + miR-122 group had the longest survival time.

## Discussion

In this study, we demonstrated that SOX2OT was up-regulated in glioma tissues and cell lines, and SOX2OT expression increased as the pathological grade increased. SOX2OT knockdown inhibited the proliferation, migration and invasion of GSCs, and promoted GSCs apoptosis. In contrast, miR-194-5p and miR-122 were downregulated in glioma tissues and cell lines. Over-expression of miR-194-5p and miR-122 inhibited the proliferation, migration and invasion of GSCs and promoted GSCs apoptosis. Silencing SOX2OT increased the expression of miR-194-5p and miR-122. Further, SOX2OT targeted miR-194-5p and miR-122 in a sequence-specific manner. SOX3 and TDGF-1 was upregulated in glioma tissues and cell lines and their expression increased as the pathological grade increased. Silencing SOX3 inhibited the proliferation, migration and invasion of GSCs, promoted GSCs apoptosis, and reduced the expression of TDGF-1 by directly binding to the TDGF-1 promoter. Over-expression of miR-194-5p and miR-122 decreased the expression of SOX3 by directly binding to the 3′UTR of SOX3. Silencing TDGF-1 expression in GSCs inhibited proliferation, migration and invasion of GSCs, and promoted GSCs apoptosis by inhibiting the JAK/STAT signaling pathway. Silencing SOX3 inhibited the expression of SOX2OT by directly binding to SOX2OT, which forms a positive feedback loop. This study demonstrated that the SOX2OT-miR-194-5p/miR-122-SOX3-TDGF-1 pathway forms a positive feedback loop, which plays an important role in regulating the biological behaviors of GSCs.

In recent years, lncRNA have been demonstrated to play an important role in tumor progression and thus has attracted an increasing amount of attention. Some lncRNAs have become biomarkers for the diagnosis, treatment and prognosis of different tumors [45–47]. This study demonstrated that SOX2OT was highly expressed in glioma tissues, U87 and U251 cell lines and GSCs, and SOX2OT expression increased with increased pathological grade of the gliomas. Silencing SOX2OT inhibited the proliferation, migration and invasion of GSCs, and promoted GSCs apoptosis, which suggested that SOX2OT may play an oncogenic role. Similarly, Liu S et al. found that SOX2OT was increased in colorectal cancer tissues and cell lines, and its high expression level was associated with the malignant progression of colorectal cancer patients. Decreased SOX2OT expression inhibited proliferation, migration, invasion and epithelial-mesenchymal transition (EMT) [16]. SOX2OT is increased in gastric cancer tissues and cells, and has become a biological marker of poor prognosis for gastric cancer [20, 48]. SOX2OT is also increased in hepatocellular carcinoma and lung cancer and acts as an oncogene, and SOX2OT may play an important role in promoting the development of esophageal squamous cell carcinoma and hepatocellular carcinoma [19, 21].

This study further confirmed that the expression of miR-194-5p and miR-122 was decreased in glioma tissues and GSCs, and the expression decreased as the pathological grade increased. Over-expression of miR-194-5p or miR-122 inhibited GSCs proliferation, migration and invasion, and promoted GSCs apoptosis; Knockdown of miR-194-5p or miR-122 produced the opposite effect. These results suggested that miR-194-5p and miR-122 act as tumor suppressor in GSCs. Recently, the role of miR-194-5p in tumors has attracted an increasing amount of attention. Here are some similar reports. Enhanced expression of miR-194-5p by exogenous miR-194-5p expression re-sensitized cells to differentiation and apoptosis [49]. MiR-194-5p is decreased in side population cells in human primary hepatocellular carcinoma, and regulated the proliferation, clone formation, anti-apoptosis, self-renewal and invasion abilities of side population cells [50]. Over-expression of miR-194-5p increased the expression of E-cadherin and inhibited the migration and invasion of colorectal cancer cells [51]. The above studies demonstrated that miR-194-5p acts as tumor suppressor gene in gliomas, acute myeloid leukemia, hepatocellular carcinoma and colorectal cancer. Moreover, miR-122 was decreased in gastric cancer tissues and cells, and miR-122 over-expression inhibited the proliferation, migration and invasion of gastric cancer cells [52]. MiR-122 is decreased in HBV-related hepatocellular carcinoma, and its expression is negatively correlated with tumor size, lymph node metastasis, TNM stage, histological type, and cell differentiation [53]. A kind of graphene-P-gp loaded with miR-122-InP@ZnS quantum dots nanocomposites induced drug-resistant liver tumor cells apoptosis [54]. Combined with the effect of miR-122 on GSCs in this study, we suggest that miR-122 may act as a tumor suppressor in gastric cancer, liver cancer and glioma.

We found that SOX2OT might harbor a binding site for miR-194-5p and miR-122 using a bioinformatics database (DIANA-LncBase). To verify this prediction, a dual-luciferase reporter assay was conducted, which demonstrated that SOX2OT could bind to miR-194-5p and miR-122. Moreover, silencing SOX2OT increased the expression of miR-194-5p and miR-122, and knockdown of SOX2OT inhibited the proliferation, migration and invasion of GSCs, and promoted apoptosis by up-regulating the expression of miR-194-5p and miR-122. Similar to our study, other reports regarding the effect of lncRNAs regulation of miRNAs expression on the biological behavior of glioma cells have been published. CRNDE promotes malignant biological behavior of glioma cells by decreasing miR-384 expression [55]. Decreased XIST expression inhibited proliferation, migration and invasion, and promoted apoptosis of GSCs through binding and up-regulation of miR-152 [56]. LncRNAs can also be used as the miRNAs sponge, and act as competing endogenous RNA, which affects regulation of miRNA target genes. HOTAIR acts as a miR-148a sponge and positively regulates Snail2 expression, which promotes cell invasion, metastasis and EMT in esophageal cancer [57]. In pancreatic cancer cells, HOTAIR plays the role of ceRNA, combines with miR-613, increased the expression of Notch3, and promoted pancreatic cancer cell proliferation, migration and invasion [58].

Previously, researchers have found that the transcription factor SOX3 of the SOX family was highly expressed in small cell lung cancer tissues [59]. SOX3 was increased in epithelial ovarian cancer, and promoted proliferation, migration and invasion and inhibited apoptosis of cancer cells [32]. Our study demonstrated that SOX3 was increased in glioma tissues and GSCs, and increased with an increase in glioma grade. Silencing SOX3 expression inhibited proliferation, migration and invasion of GSCs and promoted GSCs apoptosis. These results suggested that SOX3 may act as an oncogene in glioma and GSCs. Based on predictions of bioinformatics software (microRNA.org), a dual-luciferase reporter assay demonstrated that miR-194-5p and miR-122 could directly targeting the SOX3 3′-UTR. Moreover, in this study, we found that over-expression of miR-194-5p or miR-122 inhibited proliferation, migration and invasion of GSCs, and promoted GSCs apoptosis. Over-expression of SOX3 promoted the proliferation, migration and invasion of GSCs, and inhibited GSCs apoptosis. In addition, over-expression of SOX3 reversed the inhibitory effects of miR-194-5p and miR-122 in GSCs. These results suggested that over-expression of miR-194-5p and miR-122 can inhibit malignant biological behaviors of GSCs by directly down-regulating SOX3. Decreasing the expression of SOX2OT or increasing the expression of miR-194-5p or miR-122 inhibited SOX3 expression. Down-regulation of the expression of miR-194-5p or miR-122 reversed the inhibitory effects of SOX2OT knockdown on SOX3 expression, which indicated that SOX2OT acts as a miR-194-5p or miR-122 sponge, and thus influences the biological behaviors of GSCs.

TDGF-1 acts as an oncogene in some tumors. For example, TDGF-1 can promote EMT, migration and invasion of prostate cancer cells by activating the Wnt/β-catenin signaling pathway [60]. High expression of TDGF-1 is correlated with poor survival of prostate cancer patients. TDGF-1 and its signaling partner glucose-regulated protein 78 (GRP78) play a functional role in prostate cancer metastasis [61]. TDGF-1 expression was increased in hepatocellular carcinoma, which was associated with poor prognosis in patients subgroups stratified by tumor size, tumor differentiation, TNM, tumor recurrence and prognosis [62]. Our present study demonstrated that TDGF-1 was increased in glioma tissues and GSCs, and the expression increased as the glioma grade increased. Knockdown of TDGF-1 inhibited proliferation, migration and invasion of GSCs, and promoted GSCs apoptosis. These results suggested that TDGF-1 act as oncogene in GSCs. The high expression of TDGF-1 in glioma tissues and GSCs was consistent with that reported by Pilgaard in glioblastoma multiforme tissue and blood; it was found that high TDGF-1 expression was significantly correlated with shorter overall survival [41]. The results of this study further clarify the effect of TDGF-1 on the biological behaviors of GSCs. It has been reported that the members of the SOX family can be combined with the downstream target gene promoter region “AACAAAG” to regulate the transcription of target genes [63, 64]. In this study, we found that the promoter region of TDGF-1 contained the binding sequence of SOX3, and ChIP was used to demonstrate that SOX3 binds to the TDGF-1 promoter region and regulates TDGF-1 transcription. Further studies showed that over-expression of miR-194-5p or miR-122 decreased the expression of TDGF-1; inhibited the proliferation, migration and invasion and promote apoptosis of GSCs. Over-expression of SOX3 increased the expression of TDGF-1; promoted the proliferation, migration and invasion of GSCs and inhibited GSCs apoptosis. Moreover, over-expression of SOX3 reversed the effect of over-expression of miR-194-5p or miR-122 in GSCs. The above results showed that over-expression of miR-194-5p or miR-122 negatively regulated the expression of SOX3, which affects the transcription and expression of the target gene TDGF-1 and then inhibits the biological behavior of GSCs.

Interestingly, this study also found that the promoter region of SOX2OT also contains the SOX3 binding sequence, and ChIP assay confirmed that SOX3 could bind to the promoter region of SOX2OT. Silencing SOX3 expression remarkbly decreased the expression of SOX2OT in GSCs. SOX3 and SOX2OT were highly expressed in glioma tissues and GSCs. These results indicated that SOX3 can regulate SOX2OT upstream, which forms a positive feedback loop that regulates the biological behaviors of GSCs. Similarly, Teng et al. have found that RUNX1 can regulate the promoter activities and expression of HCP5, which indicated a positive feedback loop that regulated the biological behavior of glioma cells [65].

JAK is a non-transmembrane tyrosine kinase, that couples with cell membrane receptors. JAK kinase is phosphorylated and activated by binding of cytokines to the corresponding receptor, and phosphorylate the signaling molecules and transcriptional activator STAT, which further combines with target genes in the nucleus to play a role in signal transduction. Activation of the JAK/STAT signaling pathway can promote the proliferation, migration, invasion and other biological behaviors of tumor cells. Previous studies found that miR-294 promoted cellular proliferation and motility through the JAK/STAT pathway in bladder cancer [66]. Activation of the JAK/STAT pathway promoted the growth of pancreatic ductal adenocarcinoma cells [67]. Silencing protein kinase CK2 decreases adhesion and migration of glioma cells by suppressing activation of the JAK/STAT pathway and promotes survival of mice with intracranial human glioblastoma xenografts [68]. Our present study demonstrated that over-expression of TDGF-1 increased the expression of p-JAK/JAK and p-STAT/STAT, suggesting that TDGF-1 promotes the activity of the JAK/STAT pathway. Moreover, over-expression of TDGF-1 promoted proliferation, migration and invasion of GSCs, and inhibited GSCs apoptosis. These results suggest that TDGF-1 can regulate the biological behavior of GSCs through the JAK/STAT pathway.

Finally, the in vivo study demonstrated that SOX2OT knockdown, miR-194-5p over-expression, miR-122 over-expression and the combination of the above significantly inhibited GSCs tumor volume and prolonged survival time. Compared with the SOX2OT knockdown group, miR-194-5p over-expression or miR-122 over-expression groups, the group with the three treatments combined exhibited the lowest tumor volume and the longest survival time in nude mice. The results indicated that the combination of SOX2OT knockdown, miR-194-5p over-expression and miR-122 over-expression has potential clinical value.

## Conclusions

**Fig. 9:**
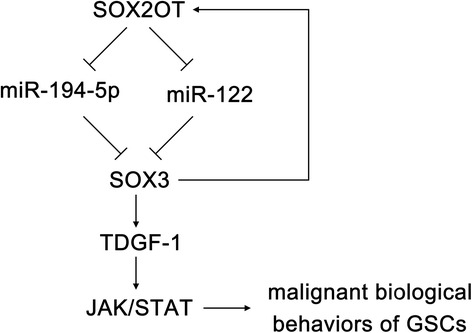
The schematic diagram of the oncogenic role of SOX2OT in GSCs

Our study revealed that SOX2OT can down-regulate the expression of SOX3 by regulating miR-194-5p and miR-122. SOX3 transcriptionally activates the expression of TDGF-1, which affect the biological behaviors of GSCs through the JAK/STAT pathway. The SOX2OT-miR-194-5p/miR-122-SOX3-TDGF-1 feedback loop plays an important role in regulating GSCs biological behaviors. Based on these findings, a functional model was proposed to illustrate the mechanism of SOX2OT knockdown on GSCs (Fig. 9). The results of this study provide a new basis for studying the mechanism of the occurrence and development of glioma, and provide a new strategy for glioma treatment.

## Additional file

Additional file 1: Figure S1. The expression of SOX2OT after cells transfection with four short-harpin plasmids of SOX2OT (SOX2OT-Homo-190, SOX2OT-Homo-456, SOX2OT-Homo-641, SOX2OT-Homo-847). **Figure S2.** The expression and effects of SOX2OT in GSC-GBM. **Figure S3.** MiR-194-5p and miR-122 exerted tumor-suppressive functions in GSC-GBM. **Figure S4.** SOX3 and TDGF-1 played oncogenic roles in GSC-GBM. **Figure S5.** SOX3 mediated tumor-suppressive effects of miR-194-5p and miR-122. (DOCX 5068 kb)
