# Correction: Knockdown of SOX2OT inhibits the malignant biological behaviors of glioblastoma stem cells via up-regulating the expression of miR-194-5p and miR-122

**DOI:** 10.1186/s12943-022-01673-y

**Published:** 2022-10-22

**Authors:** Rui Su, Shuo Cao, Jun Ma, Yunhui Liu, Xiaobai Liu, Jian Zheng, Jiajia Chen, Libo Liu, Heng Cai, Zhen Li, Lini Zhao, Qianru He, Yixue Xue

**Affiliations:** 1 Department of Neurobiology, College of Basic Medicine, China Medical University, Shenyang, 110122 People’s Republic of China; 2 Key Laboratory of Cell Biology, Ministry of Public Health of China, and Key Laboratory of Medical Cell Biology, Ministry of Education of China, China Medical University, Shenyang, 110122 People’s Republic of China; 3 Department of Neurosurgery, Shengjing Hospital of China Medical University, Shenyang, 110004 People’s Republic of China; 4 Liaoning Research Center for Translational Medicine in Nervous System Disease, Shenyang, 110004 People’s Republic of China; 5 Key Laboratory of Neuro-oncology in Liaoning Province, Shenyang, 110004 People’s Republic of China

**Correction: Mol Cancer 16, 171 (2017)**

**https://doi.org/10.1186/s12943-017-0737-1**

Following publication of the original article [1], the authors identified minor errors in Figs. 6 and 7; specifically:

- Fig. 6c: the migration assay of GSC-U251 Agomir-194-5p+SOX3(+)NC has been replaced with the correct photograph (row 2, column 4)
- Fig. 6f: the migration assay of GSC-U87 Agomir-122_SOX3(+)NC has been replaced with the correct photograph (row 1, column 3)
- Fig. 6f: the migration assay of GSC-U251 Control has been replaced with the correct photograph (row 2, column 1)
- Fig. 7f: the migration assay of GSC-U87 TDGF-1(+)NC has been replaced with the correct photograph (row 1, column 2)

The authors provided the Journal with the original data files. The corrected figure is provided here. The corrections do not have any effect on the results or conclusions of the paper.

**Fig. 6:**
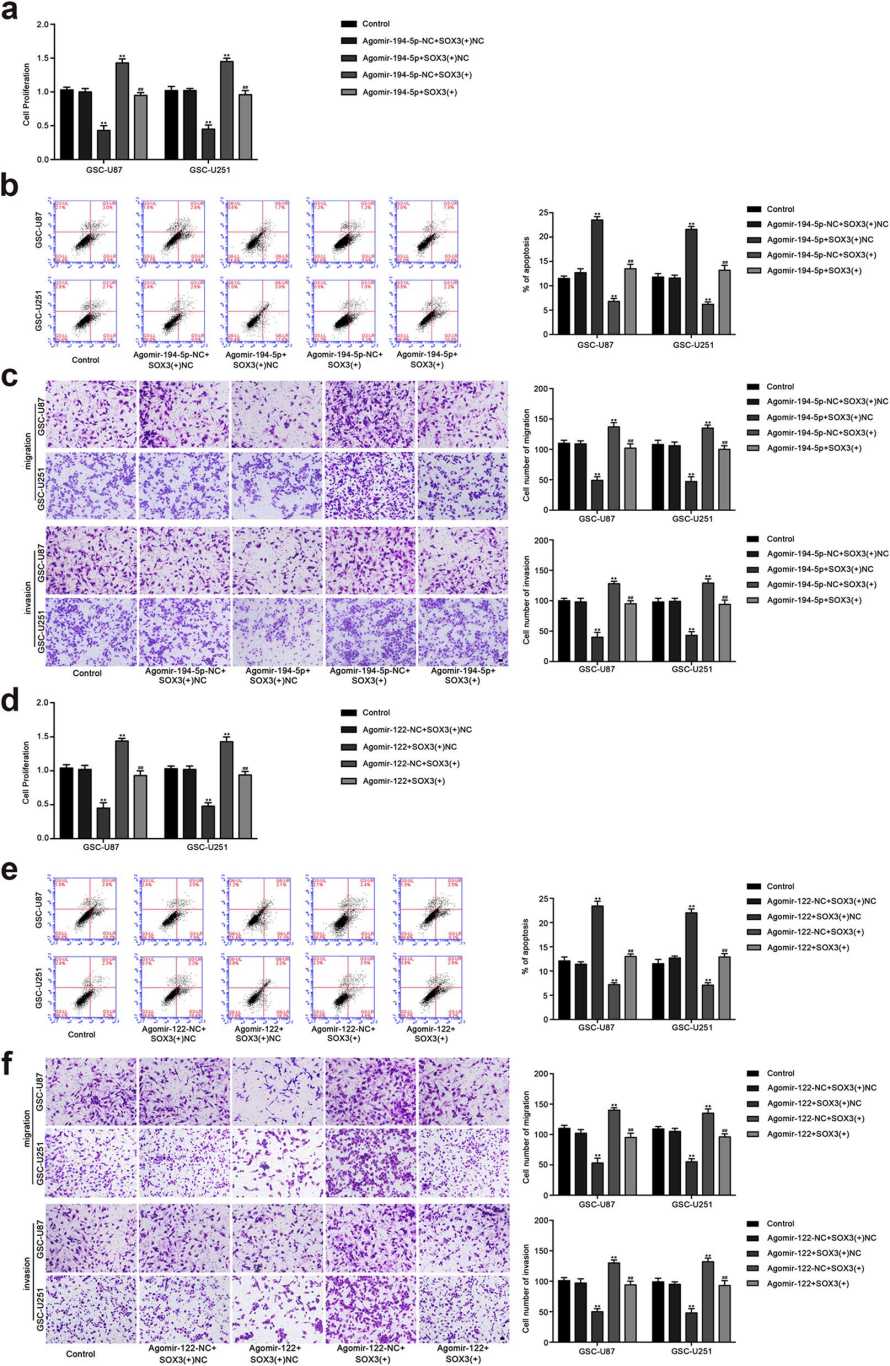
SOX3 mediated tumor-suppressive effects of miR-194-5p and miR-122. **a** CCK8 assay to evaluate the effect of miR-194-5p and SOX3 on cell proliferation of GSC-U87 and GSC-U251 cells. **b** Flow cytometry analysis to evaluate the effect of miR-194-5p and SOX3 on cell apoptosis of GSC-U87 and GSC-U251 cells. **c** Transwell assay to evaluate the effect of miR-194-5p and SOX3 on the cell migration and invasion of GSC-U87 and GSC-U251 cells. Data are presented as the mean ± SD (n = 5, each group). Scale bars represent 40 μm. ^**^ *P* < 0.01 vs. AgomiR-194-5p-NC + SOX3(+)NC group, ^##^ *P* < 0.01 vs. AgomiR-194-5p + SOX3(+)NC group. **d** CCK8 assay to evaluate the effect of miR-122 and SOX3 on cell proliferation of GSC-U87 and GSC-U251 cells. **e** Flow cytometry analysis to evaluate the effect of miR-122 and SOX3 on cell apoptosis of GSC-U87 and GSC-U251 cells. **f** Transwell assay to evaluate the effect of miR-122 and SOX3 on the cell migration and invasion of GSC-U87 and GSC-U251 cells. Data are presented as the mean ± SD (n = 5, each group). Scale bars represent 40 μm. ^**^ *P* < 0.01 vs. AgomiR-122-NC + SOX3(+)NC group, ^##^ *P* < 0.01 vs. AgomiR-122 + SOX3(+)NC group

**Fig. 7:**
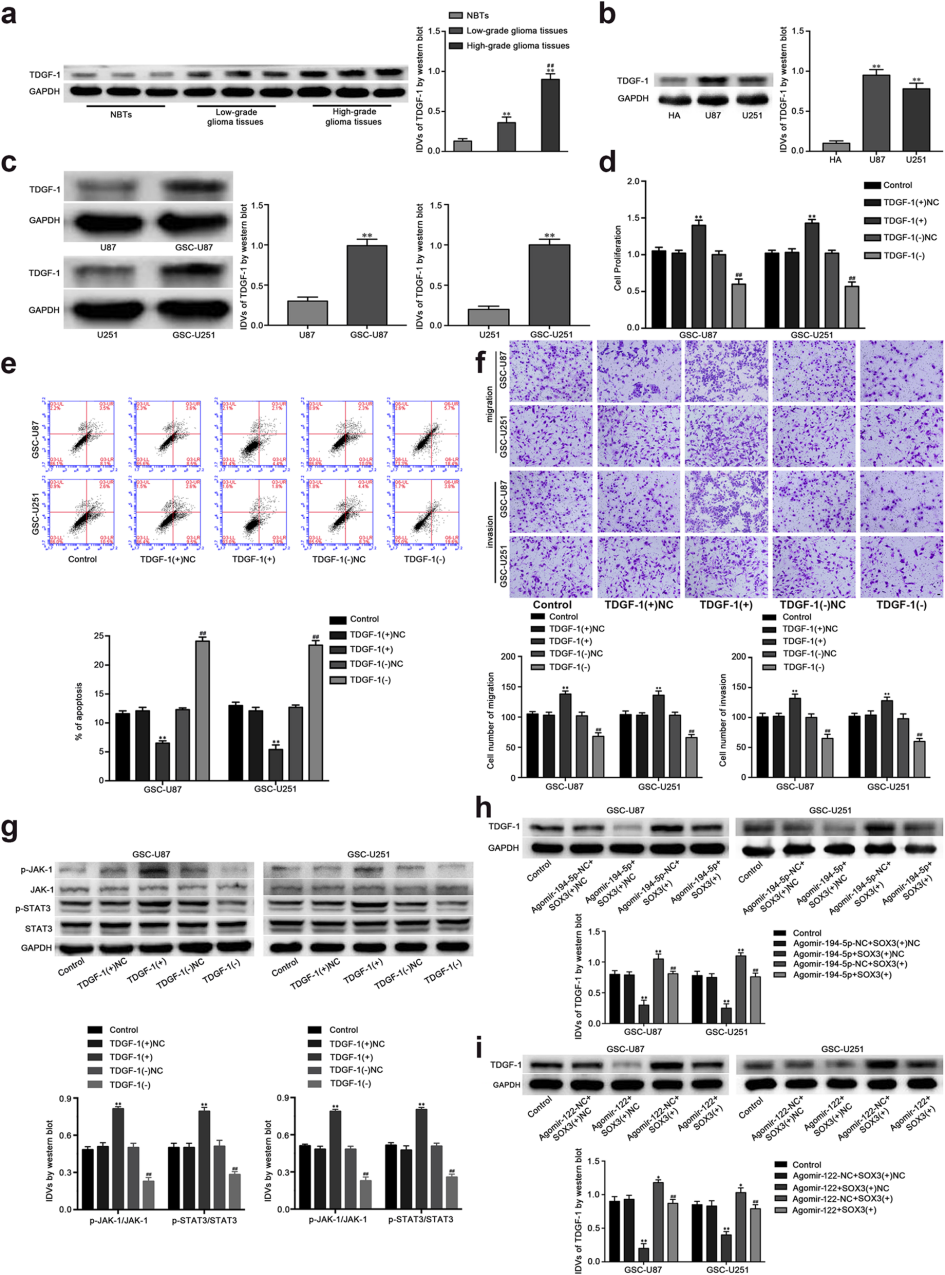
TDGF-1 endogenous expression and effect on proliferation, migration, invasion and apoptosis of GSCs. **a** TDGF-1 protein expression levels in normal brain tissues (NBTs), low-grade glioma tissues (WHO I-II) and high-grade glioma tissues (WHO III-IV) are shown. Data are presented as the mean ± SD (*n* = 3, each group). **b** The expression of TDGF-1 in human astrocytes (HA) and glioblastoma cell lines (U87 and U251). **c** The expression of TDGF-1 in glioblastoma cell lines (U87 and U251) and glioblastoma stem cells (GSC-U87, GSC-U251). **d** CCK-8 assay was used to measure the effect of TDGF-1 on the proliferation of GSC-U87 and GSC-U251 cells. **e** The apoptotic percentages of GSC-U87 and GSC-U251 were detected after TDGF-1 over-expression or knockdown. **f** Transwell assays were used to measure the effect of TDGF-1 on cell migration and invasion of GSC-U87 and GSC-U251 cells. Data are presented as the mean ± SD (n = 5, each group). ^**^ *P* < 0.01 vs. TDGF-1(+)NC, ^##^ *P* < 0.01 vs. TDGF-1(−)NC. **g** Western blot assay of the p-JAK-1/JAK-1 and p-STAT3/STAT3 expression regulated by TDGF-1. Data are presented as the mean ± SD (n = 5, each group). ^**^ *P* < 0.01 vs. TDGF-1(+)NC, ^##^ *P* < 0.01 vs. TDGF-1(−)NC. **h** Weatern blot assay were used to detect the TDGF-1 expression regulated by miR-194-5p and SOX3. Data are presented as the mean ± SD (n = 5, each group). ^**^ *P* < 0.01 vs. AgomiR-194-5p-NC + SOX3(+)NC group, ^##^ *P* < 0.01 vs. AgomiR-194-5p + SOX3(+)NC group. **i** Weatern blot assay were used to detect the TDGF-1 expression regulated by miR-122 and SOX3. Data are presented as the mean ± SD (n = 5, each group). ^**^ *P* < 0.01 vs. AgomiR-122-NC + SOX3(+)NC group, ^##^ *P* < 0.01 vs. AgomiR-122 + SOX3(+)NC group
