# Supplementary material for: Knockdown of SOX2OT inhibits the malignant biological behaviors of glioblastoma stem cells via up-regulating the expression of miR-194-5p and miR-122
Source: Mol Cancer. 2017 Nov 13;16:171. doi: 10.1186/s12943-017-0737-1 (PMC5683208; doi:10.1186/s12943-017-0737-1)
Supplement: Additional file 1: Figure S1. — The expression of SOX2OT after cells transfection with four short-harpin plasmids of SOX2OT (SOX2OT-Homo-190, SOX2OT-Homo-456, SOX2OT-Homo-641, SOX2OT-Homo-847). Figure S2. The expression and effects of SOX2OT in GSC-GBM. Figure S3. MiR-194-5p and miR-122 exerted tumor-suppressive functions in GSC-GBM. Figure S4. SOX3 and TDGF-1 played oncogenic roles in GSC-GBM. Figure S5. SOX3 mediated tumor-suppressive effects of miR-194-5p and miR-122. (DOCX 5068 kb) [file 12943_2017_737_MOESM1_ESM.docx]

**
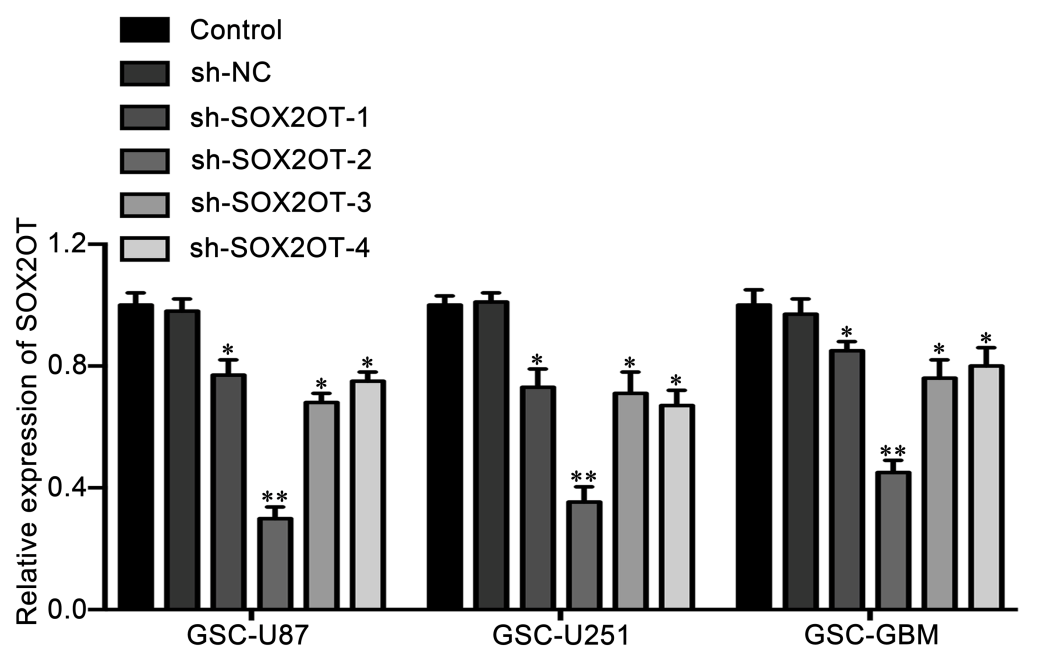
**

**Additional file 1: Figure S1 The expression of SOX2OT after cells transfection with four short-harpin plasmids of SOX2OT (SOX2OT-Homo-190, SOX2OT-Homo-456, SOX2OT-Homo-641, SOX2OT-Homo-847).**

Data are presented as the mean±SD (n=5,each group). **P*<0.05 vs. sh-NC group; ***P*<0.01 vs. sh-NC group.

**
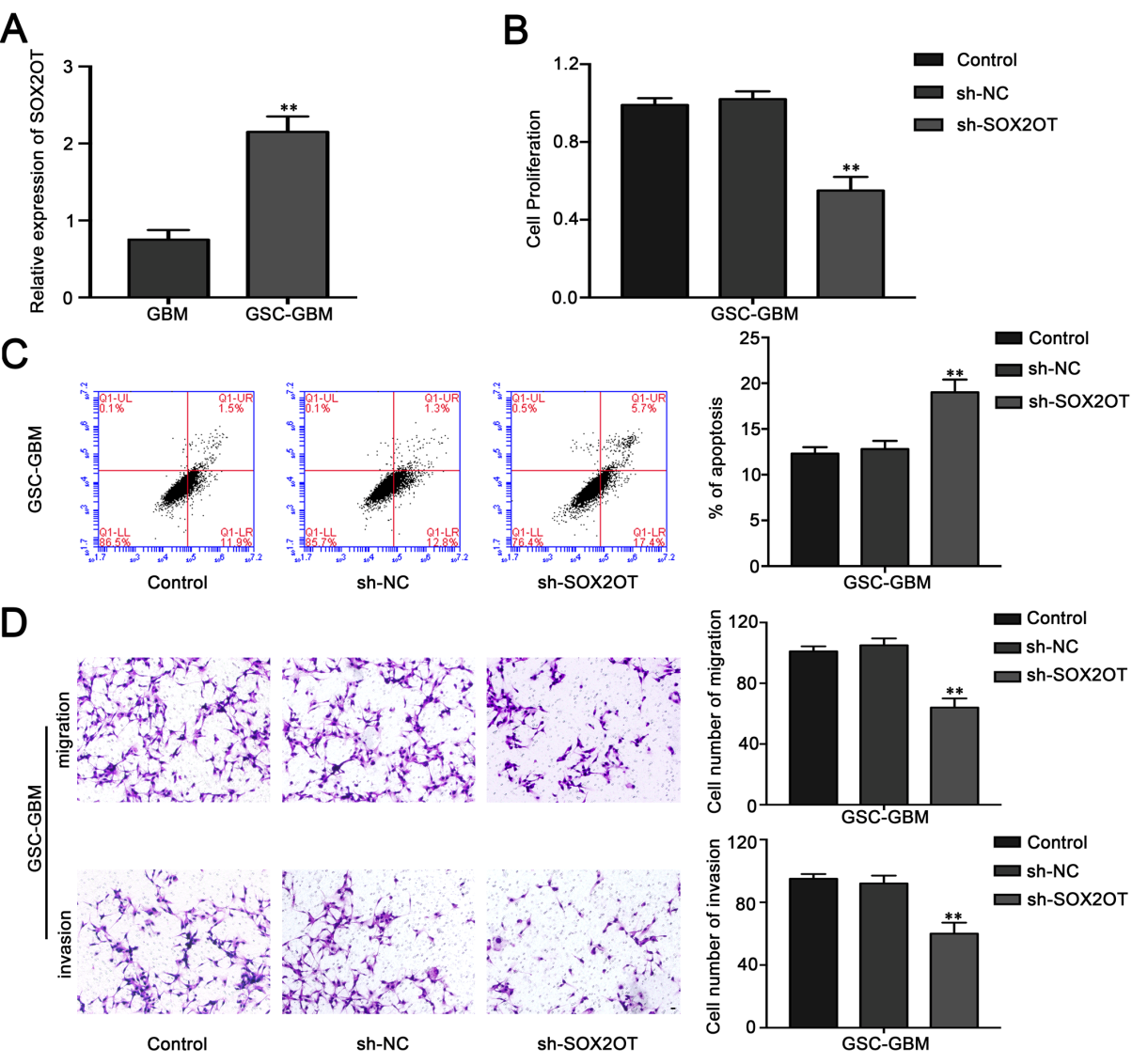
**

**Additional file 1: Figure S2 The expression and effects of SOX2OT in GSC-GBM.**

(A) The expression of SOX2OT in derived cells of glioblastoma multiforme (GBM) and GBM stem cells (GSC-GBM). Data are presented as the mean±SD(n=5, each group). ***P*<0.01 vs. GBM group. (B) CCK-8 assay was used to measure the effect of SOX2OT on the proliferation of GSC-GBM. (C)The apoptotic percentages of GSC-GBM were detected after SOX2OT knockdown. (D) Transwell assays were used to measure the effect of SOX2OT on cell migration and invasion of GSC-GBM. Data are represented as the mean±SD (n=5, each group). ***P*<0.01 vs. sh-NC group. Scale bars represent 40μm.

**
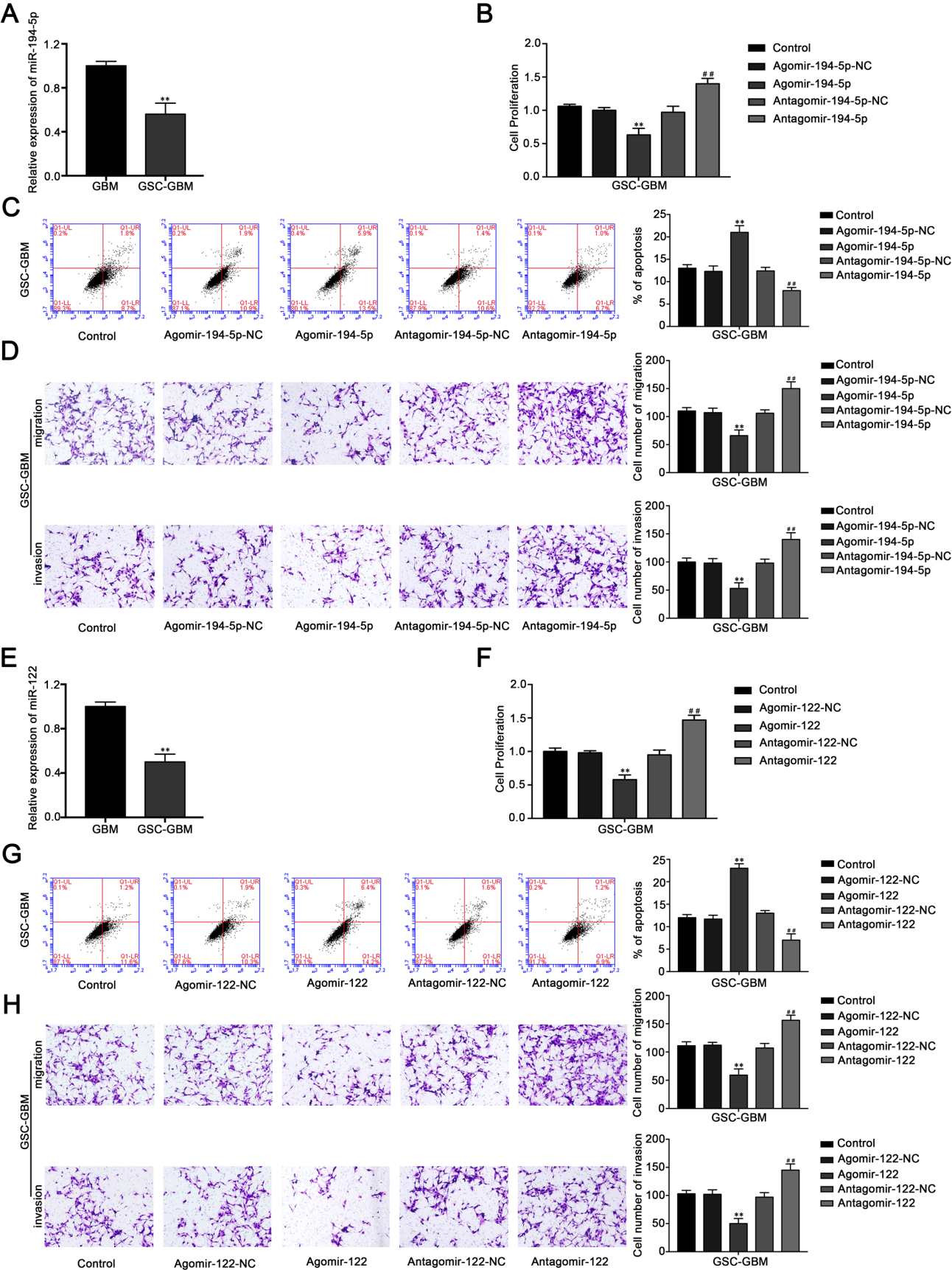
**

**Additional file 1: Figure S3 MiR-194-5p and miR-122 exerted tumor-suppressive functions in GSC-GBM.**

(A) The expression of miR-194-5p in GBM and GSC-GBM. Data are presented as the mean±SD (n=5, each group).***P*<0.01 vs. GBM group. (B) CCK-8 assay was used to measure the effect of miR-194-5p on the proliferation of GSC-GBM. (C)The apoptotic percentages of GSC-GBM were detected after miR-194-5p over-expression or inhibition. (D) Transwell assays were used to measure the effect of miR-194-5p on cell migration and invasion of GSC-GBM. Data are represented as the mean±SD (n=5, each group). ***P*<0.01 vs. Agomir-194-5p-NC group, ^##^*P*<0.01 vs. Antagomir-194-5p-NC group. (E) The expression of miR-122 in GBM and GSC-GBM. Data are presented as the mean±SD (n=5, each group). ***P*<0.01 vs. GBM group. (F) CCK-8 assay was used to measure the effect of miR-194-5p on the proliferation of GSC-GBM. (G)The apoptotic percentages of GSC-GBM were detected after miR-122 over-expression or inhibition. (H) Transwell assays were used to measure the effect of miR-122 on cell migration and invasion of GSC-GBM. Data are represented as the mean±SD (n=5, each group). ***P*<0.01 vs. Agomir-122-NC group, ^##^*P*<0.01 vs. Antagomir-122-NC group. Scale bars represent 40μm.


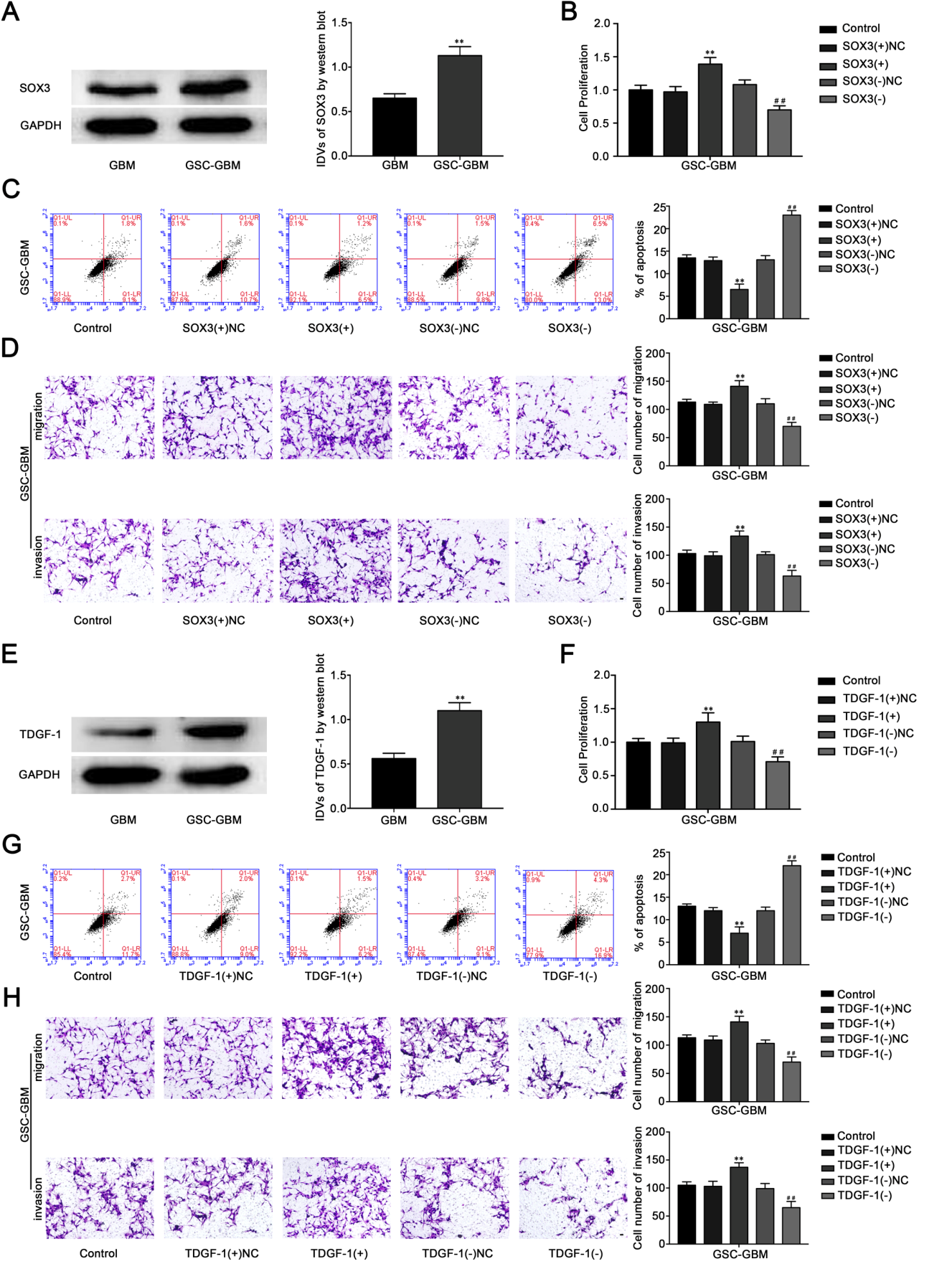


**Additional file 1: Figure S4 SOX3 and TDGF-1 played oncogenic roles in GSC-GBM.**

(A) The protein expression level of SOX3 in GBM and GSC-GBM. Data are presented as the mean±SD (n=5, each group). ***P*<0.01 vs. GBM group. (B) CCK-8 assay was used to measure the effect of SOX3 on the proliferation of GSC-GBM. (C)The apoptotic percentages of GSC-GBM were detected after SOX3 over-expression or knockdown. (D) Transwell assays were used to measure the effect of SOX3 on cell migration and invasion of GSC-GBM. Data are represented as the mean±SD (n=5, each group). ***P*<0.01 vs. SOX3(+)NC group, ^##^*P*<0.01 vs. SOX3(-)NC group. (E) The protein expression level of TDGF-1 in GBM and GSC-GBM. Data are presented as the mean±SD(n=5, each group).***P*<0.01 vs. GBM group. (F) CCK-8 assay was used to measure the effect of TDGF-1 on the proliferation of GSC-GBM. (G)The apoptotic percentages of GSC-GBM were detected after TDGF-1 over-expression or knockdown. (H) Transwell assays were used to measure the effect of TDGF-1 on cell migration and invasion of GSC-GBM. Data are represented as the mean±SD (n=5, each group). ***P*<0.01 vs. SOX3(+)NC group, ^##^*P*<0.01 vs. SOX3(-)NC group. Scale bars represent 40μm.


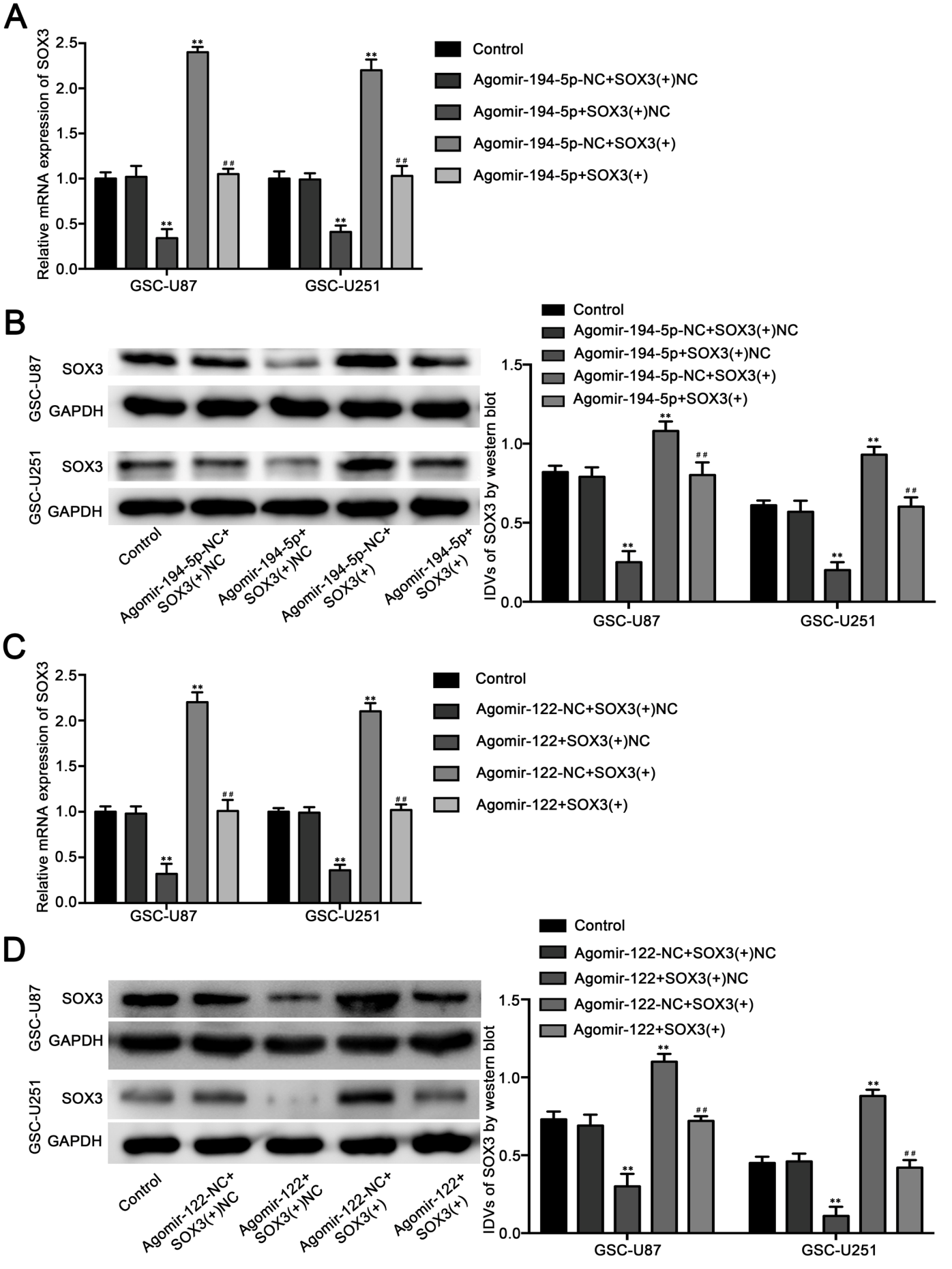


**Additional file 1: Figure S5 SOX3 mediated tumor-suppressive effects of miR-194-5p and miR-122.**

(A) Real-time PCR and (B) western blot assays were used to detect the SOX3 expression regulated by miR-194-5p and SOX3. Data are represented as the mean±SD (n=5, each group). ***P*<0.01 vs. Agomir-194-5p-NC+SOX3(+)NC group; ^##^*P*<0.01 vs. Agomir-194-5p+SOX3(+)NC group. (C) Real-time PCR and (D) western blot assays were used to detect the SOX3 expression regulated by miR-122 and SOX3. Data are represented as the mean±SD (n=5, each group). ***P*<0.01 vs. Agomir-122-NC+SOX3(+)NC group; ^##^*P*<0.01 vs. Agomir-122+SOX3(+)NC group.
